# Supplementary material for: Elements Characterising Multicomponent Interventions Used to Improve Disease Management Models and Clinical Pathways in Acute and Chronic Heart Failure: A Scoping Review
Source: Healthcare (Basel). 2023 Apr 25;11(9):1227. doi: 10.3390/healthcare11091227 (PMC10178532; doi:10.3390/healthcare11091227)
Supplement: Supplementary file 1 [file healthcare-11-01227-s001.zip › healthcare-2305652-supplementary.pdf]

Supplementary Table S1: Preferred Reporting Items for Systematic reviews and Meta-Analyses extension for Scoping Reviews (PRISMA-ScR) Checklist

SECTION	ITEM	PRISMA-ScR CHECKLIST ITEM	REPORTED ON PAGE #
**TITLE**			
Title	1	Identify the report as a scoping review.	p.1
**ABSTRACT**			
Structured summary	2	Provide a structured summary that includes (as applicable): background, objectives, eligibility criteria, sources of evidence, charting methods, results, and conclusions that relate to the review questions and objectives.	p.1
**INTRODUCTION**			
Rationale	3	Describe the rationale for the review in the context of what is already known. Explain why the review questions/objectives lend themselves to a scoping review approach.	p.2
Objectives	4	Provide an explicit statement of the questions and objectives being addressed with reference to their key elements (e.g., population or participants, concepts, and context) or other relevant key elements used to conceptualize the review questions and/or objectives.	p.2
**METHODS**			
Protocol and registration	5	Indicate whether a review protocol exists; state if and where it can be accessed (e.g., a Web address); and if available, provide registration information, including the registration number.	p.3
Eligibility criteria	6	Specify characteristics of the sources of evidence used as eligibility criteria (e.g., years considered, language, and publication status), and provide a rationale.	p.3
Information sources*	7	Describe all information sources in the search (e.g., databases with dates of coverage and contact with authors to identify additional sources), as well as the date the most recent search was executed.	p.3-4
Search	8	Present the full electronic search strategy for at least 1 database, including any limits used, such that it could be repeated.	Supplementary table 2
Selection of sources of evidence†	9	State the process for selecting sources of evidence (i.e., screening and eligibility) included in the scoping review.	p.4
Data charting process‡	10	Describe the methods of charting data from the included sources of evidence (e.g., calibrated forms or forms that have been tested by the team before their use, and whether data charting was done independently or in duplicate) and any processes for obtaining and confirming data from investigators.	p.4
Data items	11	List and define all variables for which data were sought and any assumptions and simplifications made.	p.4
Critical appraisal of individual sources of evidence§	12	If done, provide a rationale for conducting a critical appraisal of included sources of evidence; describe the methods used and how this information was used in any data synthesis (if appropriate).	N/A
Synthesis of results	13	Describe the methods of handling and summarizing the data that were charted.	p.4
**RESULTS**			
Selection of sources of evidence	14	Give numbers of sources of evidence screened, assessed for eligibility, and included in the review, with reasons for exclusions at each stage, ideally using a flow diagram.	p.4<br>Figure 1

SECTION	ITEM	PRISMA-ScR CHECKLIST ITEM	REPORTED ON PAGE #
Characteristics of sources of evidence	15	For each source of evidence, present characteristics for which data were charted and provide the citations.	Supplementary table 3
Critical appraisal within sources of evidence	16	If done, present data on critical appraisal of included sources of evidence (see item 12).	N/A
Results of individual sources of evidence	17	For each included source of evidence, present the relevant data that were charted that relate to the review questions and objectives.	pp.5-13 & Tables 3-6 Supplementary table 3
Synthesis of results	18	Summarize and/or present the charting results as they relate to the review questions and objectives.	Tables 3-6
**DISCUSSION**			
Summary of evidence	19	Summarize the main results (including an overview of concepts, themes, and types of evidence available), link to the review questions and objectives, and consider the relevance to key groups.	p.13
Limitations	20	Discuss the limitations of the scoping review process.	p.15
Conclusions	21	Provide a general interpretation of the results with respect to the review questions and objectives, as well as potential implications and/or next steps.	p.15
**FUNDING**			
Funding	22	Describe sources of funding for the included sources of evidence, as well as sources of funding for the scoping review. Describe the role of the funders of the scoping review.	p.16

JBI = Joanna Briggs Institute; PRISMA-ScR = Preferred Reporting Items for Systematic reviews and Meta-Analyses extension for Scoping Reviews.
* Where *sources of evidence* (see second footnote) are compiled from, such as bibliographic databases, social media platforms, and Web sites.
† A more inclusive/heterogeneous term used to account for the different types of evidence or data sources (e.g., quantitative and/or qualitative research, expert opinion, and policy documents) that may be eligible in a scoping review as opposed to only studies. This is not to be confused with *information sources* (see first footnote).
‡ The frameworks by Arksey and O'Malley (6) and Levac and colleagues (7) and the JBI guidance (4, 5) refer to the process of data extraction in a scoping review as data charting.
§ The process of systematically examining research evidence to assess its validity, results, and relevance before using it to inform a decision. This term is used for items 12 and 19 instead of "risk of bias" (which is more applicable to systematic reviews of interventions) to include and acknowledge the various sources of evidence that may be used in a scoping review (e.g., quantitative and/or qualitative research, expert opinion, and policy document).

From: Tricco AC, Lillie E, Zarin W, O'Brien KK, Colquhoun H, Levac D, et al. PRISMA Extension for Scoping Reviews (PRISMAScR): Checklist and Explanation. Ann Intern Med. 2018;169:467–473. doi: 10.7326/M18-0850.

Supplementary Table S2. Search strategy

PICO(S) framework question search	
Population	Adult patients with heart failure at any stage of the disease
Intervention	Disease management interventions and clinical pathways to manage the chronic and acute phases of HF patients
Comparator	Standard care or any other model of care
Outcomes	Studies investigating any outcome of efficacy, effectiveness and costs will be considered.
Study design	All study designs were included given the broad scope of the review. No limits were given on the duration of the intervention or the length of follow-up.

Supplementary Table S3. Description of studies included.

Study included	Method study	N (intervention)	N (control)	N Total	Inclusion/ exclusion criteria	Aim	Intervention	Control	Follow-up period
Abraham et al. (2011) [30]	RCT	270	280	575	HF III	The hypothesis in the, the CardioMEMS Heart Sensor Allows Monitoring of Pressure to Improve Outcomes in NYHA Class III Heart Failure Patients (CHAMPION) trial, was that management of heart failure by use of pulmonary artery pressures would greatly reduce the rate of heart-failure related hospitalisation.	After sensor implantation, participants were randomly assigned to a treatment group, which allowed the physician access to pulmonary artery pressure readings. Patients were trained to use the home electronic console. All patients in the treatment and control group took daily pressure readings, which were transmitted via a modem to a database. Sensor data were only available to the physician for patients in the treatment group, and medication changes were made according to the sensor's haemodynamic dynamics information and the standard of care.	Usual care	15 months
Adamson et al. (2011) [31]	RCT	202	198	400	HF II-III	To evaluate the safety of combining an implantable hemodynamic monitoring system with a single chamber implantable cardioverter-defibrillator (ICD) and to prospectively evaluate the clinical efficacy of hemodynamic-guided HF management in patients with (NYHA) class II and III HF.	Each patient was taught how to use the IHM-ICD and the home device to upload information from the IHM-ICD via a telephone connection. The investigators were asked to review the haemodynamic information in the treatment group on a weekly basis and to adapt medical therapies in response to the pressure information. The patient was provided with an atmospheric pressure device (EPR). During the execution of the study, an automatic pressure change detection algorithm was implemented to simplify the visual identification of subtle pressure changes that proved to cause heart failure.	Usual care	12 months
Adlbrecht et al. (2011) [32]	RCT	85	47	190	HF III-IV LVEF <40%	To analyse prospectively costs of different treatment concepts.	Within 3 days before discharge, patients were randomized to usual care (UC), home-based nurse care (HNC) and HNC group in which decision-making was based on amino terminal pro B natriuretic peptide levels (BNC). HNC and BNC included two scheduled consultations at the outpatient heart	Usual care	9 months

							failure specialist clinic, as well as care by a specialized heart failure nurse comprising of four home visits and telephone contact. The nurse checked weight, symptoms and signs, heart rate and blood pressure, and organized blood analyses. The nurse was in charge of individualized patient and caregiver education and enhancement of self-management.		
Agren et al. (2012) [33]	RCT	71	84	155	HF II e IV	To evaluate the effects of an integrated dyad care program with education and psychosocial support to patients with CHF and their partners during a post discharge period after acute deterioration of CHF.	The sample included dyads consisting of a patient and an informal caregiver partner. Cognitive-behavioural strategies were chosen to help dyads recognise and modify factors contributing to physical and emotional distress, changing thoughts and behaviours in a positive way, and assisting dyads in problem-solving related to the implementation of self-care strategies. The intervention included HF training and was delivered in 3 sessions at the dyads' homes or in the clinic through face-to-face counselling with the nurse, a computer-based CD-ROM programme and other written training materials.	Usual care	3 and 12 months
Agren et al. (2013) [34]	RCT	71	84	155	HF II-IV	To estimate the cost-effectiveness of a nurse-led education and psychosocial support programme for patients with HF and their partners.	The patient/caregiver dyads in the experimental group participated in an integrated intervention at home or in the HF outpatient clinic. The intervention was delivered in three face-to-face sessions and included nurse-led counselling with educational, supportive, and behavioural components after discharge. The dyads were assisted by the nurse during the use of a computerised programme. Each session included HF education and the development of problem-solving skills to help the dyads recognise and modify factors contributing to psychological and emotional distress. The intervention focused on modifying thoughts and behaviours and implementing self-care strategies.	Usual care	12 weeks after discharge.

Agrinier et al. (2013) [35]	Prospective cohort study	/	/	1223	HF	To assess the effectiveness of a disease management program (DMP) in heart failure (HF) on the incidence of HF hospitalizations and related costs in a real-world population-based setting.	Educational and home-visit monitoring programs by HF-trained nurses. General physicians received automatic alerts about patients' significant clinical or biological changes.	NA	552 days
Aguado et al. (2010) [36]	RCT	42	64	106	HF II-IV LVEF <45%	To evaluate the effectiveness of a single home-based educational intervention for patients admitted with heart failure.	A physician was responsible for the training of the two nurses involved in the delivery of the educational intervention. The intervention consisted of a visit by a qualified nurse to patients in their homes one week after discharge to assess the patient and to hold an educational session focusing on self-management, habits and prevention activities.	Usual care	6 and 24 months
Agvall et al. (2013) [37]	RCT	81	79	160	HF I–IV	To evaluate if the use of HF management programmes (HFMPs) has beneficial effects on HF patients in primary healthcare (PHC).	Initial consultation with the GP and then a visit to a HF nurse, receiving oral and written information about HF from educated HF nurses as well as from a validated computer-based information programme. The HF nurse made a follow-up visit after 2 months and contacted the participants by telephone after 1 month and after 6 months. At the end of the study period (12 months), all participants attended a final visit to a physician.	Usual care	1 year
Albert et al. (2007) [38]	RCT	59	53	112	HF	To examine short-term impact of video education (VE) in addition to standard education (SE) on HF healthcare utilization and self-care behaviour adherence.	Patients in the experimental group, in addition to standard education from physicians and RNs and a handbook on heart failure, received an educational video on self-care and self-management of HF tie watch at home	Standard education	3 months
Aldamiz-Echevarr ́ıa IB et al. (2017) [39]	RCT	137	142	279	HF	To determine the effectiveness of a primarily educational intervention in heart failure (HF) patients implemented in a home care unit.	Between discharge from hospital and up to 15 days later, patients assigned to the programme group were cared for at home by doctors and nurses from the home care unit. Nurses visited each patient 2, 5 and 10 days after discharge to administer the educational programme. A printed handbook with the same information and a telephone number to be contacted in case of doubts was also handed out.	Usual care	12 months

Angermann et al. (2012) [40]	RCT	352	363	715	HF LVEF < 40%	To evaluate whether a nurse-coordinated disease management program (HeartNetCare-HF, HNC) would have a favourable impact on time to death or rehospitalization and various prespecified secondary end points in patients discharged from hospital after cardiac decompensation.	HNC included the following elements: face-to-face contact in the hospital between the specialist nurse, the patient and family members to explain the intervention, practice supervision of clinical signs and symptoms, and provide participants with educational materials; telephone monitoring by means of a standardised questionnaire on indicators of worsening HF, symptoms, medication, healthcare utilisation, mood and general health and wellbeing; up titration of medication in collaboration with GPs; specialist care; training and supervision of interveners.	Usual care	180 days
Antonicelli et al. (2008) [41]	RCT	28	29	57	HF II–IV LVEF 40%	To explore whether, as compared to standard care from a specialized CHF management team, the addition of home telemonitoring to an integrated CHF patient care system may reduce mortality and rate of re-admission to hospital in elderly CHF patients.	Subjects randomised to the intervention group (home telemonitoring), or one of their relatives, were contacted by telephone at least once a week by the heart failure team to collect information on symptoms and adherence to therapy, blood pressure, heart rate, body weight and urine output. A weekly ECG transmission was also requested. The treatment regimen was regularly re-evaluated and, whenever necessary, clinical visits were carried out based on telemonitored data or telephone interviews.	Usual care	12 months
Artinian et al. (2003) [42]	RCT	9	9	18	HF LVEF <40%	The study compared the effects of a self-care and medication compliance device, linked to a Web-based monitoring system, to the effects of usual care alone on compliance with recommended self-care behaviours; medication taking; quality of life; distance walked during a 6-minute walk test; and New York Heart Association Functional Class. Was also assessed patient experiences living	A web-based monitoring system was used, which included a medication compliance device connected via the patient's telephone line to a database accessible via the Internet. The device asked patients about their heart-healthy diet, physical activity and medication, as well as prompting answers to questions about symptoms, blood pressure and weight. Through a browser interface, clinicians were able to monitor patients, provide advice and update treatment regimes.	NA	3 months

						with the compliance device.			
Atienza et al. (2004) [43]	RCT	164	174	338	HF	To assess the effectiveness of a discharge and outpatient management program in a non-selected cohort of patients hospitalized for HF.	Patients and their families were educated about the disease before discharge through an interview with a cardiology nurse. A visit with the general practitioner was scheduled within 2 weeks after discharge. Regular follow-up visits were scheduled at the heart failure outpatient clinic every 3 months where, in addition to the routine clinical assessment, the cardiologist reviewed the patients' performance and introduced corrective strategies to improve adherence and response to treatment. The telemonitoring phase lasted from discharge until the end of the study and consisted of facilitated telephone communication with a monitor (SCT), which provided a mobile phone number to be contacted 24 hours a day.	Usual care	1 year
Austin et al. (2005) [44]	RCT	100	100	200	HF II-III LVEF ≤40%	To determine whether a cardiac rehabilitation programme improved on the outcomes of an outpatient heart failure clinic (standard care) for patients, over 60 years of age, with chronic heart failure.	8-week cardiac rehabilitation programme that was coordinated by the clinical nurse specialist. Patients attended classes twice weekly for a period of 2.5 h. Patients graduated from the programme to a 16-week community-based care regimen consisting of weekly 1-h exercise sessions supervised by an exercise instructor. During the first 8 weeks of the trial, experimental patients received additional education input, during weekly group sessions, on a variety of essential topics (medication, diet, exercise) from members of the multidisciplinary team.	Usual care	24 weeks
Baker et al. (2011) [45]	RCT	303	302	605	HF II-IV	To determine whether a "teach to goal" (TTG) educational and behavioural support program provided incremental benefits to a	Intensive goal-based education program that included the same single in-person education session plus a series of follow-up education phone calls.	Single in-person education session	2 years

						brief (1 hour) educational intervention (BEI) for knowledge, self-care behaviours, and HF-related quality of life (HFQOL).			
Balk et al. (2008) [46]	RCT	101	113	214	HF I–IV	Evaluate the effects of a remote guidance system, in patients with chronic heart failure.	The patients in the intervention group received a tele-guidance system, in addition to the scheduled visits by the cardiologist, which included a home television channel providing educational material, medication reminders, health surveys and motivational messages. A subset of patients was also provided with automatic devices for daily blood pressure and weight measurement and communicated automatically with a Medical Service Centre. Tele-guidance and monitoring of measurements were performed by nurses and supervised by physicians.	Usual care	288 days
Barnason et al. (2003) [47]	RCT	18	17	35	HF	To determine the impact of a home communication intervention (HCI) for ischemic heart failure Coronary Artery Bypass Graft (CABG) patients 65 years of age on self-efficacy, coronary artery disease risk factor modification and functioning posthospitalization.	The HCI was delivered to subjects using a device called Health Buddy that connects to the patient's phone as a means of communication and provides patients with symptom assessment and strategies for managing reported symptoms; education and strategies for modifying CAD risk factors; education on CABG recovery; positive reinforcement to increase patients' self-efficacy in relation to symptom management, functioning, and adherence to modifying CAD risk factors. Subjects' responses were downloaded onto a website; research nurses reviewed patients' responses daily to ensure that the appropriate daily HCI session was sent to the patient via Health Buddy.	Usual patient education and counselling provided prior to hospital discharge.	3 months

Bekelman et al. (2015) [48]	RCT	193	199	392	HF	To determine the effectiveness of a collaborative care patient-centred disease management (PCDM) intervention to improve the health status of patients with HF.	The intervention included multidisciplinary collaborative HF care, depression screening and treatment, and telemonitoring. The collaborative care team, consisting of a nurse coordinator, a general practitioner, a cardiologist, and a psychiatrist, reviewed the electronic medical record and baseline depression scores from the PHQ-9. Intervention patients who tested positive for depression received the depression treatment component of the intervention. The telemonitoring system collected daily measurements. Patients with depression were asked questions about mood and behaviour. The telemonitoring system assigned a risk to each system response. The nurse examined the risk indicators and decided whether an action should be taken.	Usual care	1 year
Benatar et al. (2003) [49]	RCT	108	108	216	HF III-IV LVEF 40%	To compare outcomes for patients whose home health care was provided by nurse tele management (NTM) or home nurse visits (HNV). The outcomes of interest included HF readmissions and length of stay (LOS), HF hospitalization charges, and preintervention and postintervention quality-of-life (QOL) measurements.	Patients with HF used trans telephonic home monitoring devices to measure weight, blood pressure, heart rate and oxygen saturation level and data were transmitted daily to an Internet site. An advanced practice nurse (APN) evaluates the objective data transmitted by the patient, performs telemonitoring evaluations, titrates drug therapy and performs patient education. The telemonitoring device transmitted an alarm to a pager carried by the APN if values outside the desired ranges were detected.	Usual care	12 months
Berger et al. (2010) [50]	RCT	96	90	278	HF III-IV LVEF 40%	To investigate whether the addition of N-terminal pro–B-type natriuretic peptide–guided, intensive patient management (BM) to multidisciplinary care (MC) improves outcome in patients following hospitalization due to HF.	The MC included care by a heart failure nurse, which included home visits and telephone contact, pre-programmed consultations by the heart failure specialist, and on-demand consultations that were performed if the heart failure nurse noticed a deterioration in the patient's condition. Each consultation consisted of a physical examination and a 12-lead	Usual care	12 months

							electrocardiogram was examined; laboratory tests were performed. In patients with an NT-proBNP level at discharge 2,200 pg/ml (BM), outpatient visits to a specialist were made at least every 2 weeks, in addition to the MC, to optimise medical therapy as quickly as possible.		
Bernocchi et al. (2016) [51]	RCT	60	60	120	COPD HF II-IV	To investigate the feasibility and efficacy of an integrated, home-based, medical/nursing intervention plus a rehabilitation program versus conventional care in patients with coexisting COPD/CHF.	All patients randomised to the intervention group received an educational intervention from the NT (nurse tutor) and PT (physiotherapist) and were followed by both during the home programme. Subsequently, they made a weekly telephone call collecting information on the disease status and symptoms. The patients were provided with a pulse oximeter and a portable electrocardiograph and were instructed on how to transmit the ECG. The patients were assessed by the physiotherapist, who drew up a customised rehabilitation programme to meet the specific needs of each patient.	Usual care	6 months
Bernocchi et al. (2018) [52]	RCT	56	56	112	COPD HF II-IV	To study the feasibility and efficacy of an integrated telerehabilitation home-based programme (Telereab- HBP), 4 months long, in patients with combined COPD and CHF.	The patients received an educational intervention by a nurse tutor (NT) and a physiotherapist tutor (PT) and were followed up by both during the Telereab-HBP. The NT made a weekly phone call to each patient, collecting information on disease status and symptoms, offering advice on diet, lifestyle, and medication. Patients were provided with a pulse oximeter and a portable electrocardiograph for real-time telemonitoring of vital signs. Patients could call in case of urgent or emergency needs 24 hours a day. The PT designed a customised exercise programme for each patient, which was equipped with a mini-ergometer, pedometer and diary.	Usual care	6 months
Black et al. (2014) [53]	RCT	750	750	1500	HF	To evaluate the effectiveness of a care transition intervention in reducing all-cause 180-day	The Better Effectiveness After Transition - Heart Failure (BEAT-HF) intervention consists of three components: heart failure education	Usual care	6 months

						hospital readmissions for older adults hospitalized with heart failure.	before discharge, periodic telephone coaching and home telemonitoring. Pre-discharge health education is conducted with the "teach-back" approach by the practice nurse and guides the patients through a brochure. Telephone nursing care then takes place on a weekly basis during the first month after discharge.		
Blue et al. (2001) [54]	RCT	84	81	165	HF II-IV	To determine whether nurse intervention, when used in addition to routine care, can reduce the morbidity and mortality related to chronic heart failure.	The intervention consisted of a series of planned home visits at decreasing frequency, supplemented by telephone contacts as needed. The aim was to educate the patient about heart failure and its treatment, optimise therapy (medication, diet, exercise), monitor electrolyte concentrations, teach self-monitoring and management, maintain contact with other health and social workers if necessary, and provide psychological support. Before the start of the study, the nurses received training in these roles.	Usual care	12 months
Bourge et al. (2008) [55]	RCT	134	140	274	HF III-IV	To determine whether a heart failure (HF) management strategy using continuous intracardiac pressure monitoring could decrease HF morbidity.	Study participants were implanted with the ICHM, an implantable continuous haemodynamic monitor for ambulatory HF management, which continuously measures and stores haemodynamic information. All patients were instructed to transmit information from the ICHM at least weekly using a home monitor interrogating the device. Physicians could access the transmitted data on the ICHM website. During follow-up, physicians reviewed the haemodynamic information of the intervention group at least weekly to determine volume status. Patients were asked to record daily weights and to document any changes made to daily medication.	Usual care	6 months

Brandon et al. (2009) [56]	RCT before-after	10	10	20	HF	To determine the effect of an advanced-practice-nurse (APN)-led telephone intervention on hospital readmissions, quality of life, and self-care behaviors (SCBs) of patients with heart failure (HF).	APN-led telephone intervention. The intervention included education about the pathophysiology of HF, a low sodium diet, smoking cessation, flu/pneumonia vaccinations, when to call the physician with symptoms of exacerbation, and medication adherence.	Usual care	6 months
Brennan et al. (2010). [57]	RCT	138	146	282	HF	Evaluation of an innovative home care nursing model, technology enhanced practice (TEP).	Either a web-computer or the modifications to computers that enabled patients to access a suite of technology tools, housed on a website, that addressed education, symptom monitoring, and communication. During home visits, nurses chose technology tools that addressed patient needs and goals and used the tools interactively with the patient, taught patients to use them independently between visits, and selectively monitored use between visits.	Usual care	1, 4, 8, 12, and 24 weeks
Brotons et al. (2009) [58]	RCT	144	139	283	HF	To determine whether a home-based intervention can reduce mortality and hospital readmissions and improve quality of life in patients with heart failure.	Before being discharged from hospital, the patients assigned to the intervention group received information on their disease and the prescribed pharmacological and non-pharmacological treatments, including a brochure containing information on heart failure. Throughout the year, nurses made monthly visits to the patients' homes and contacted them by telephone every 15 days to assess their clinical status. Each home visit included education about the disease and recognition of warning symptoms, assessment of adherence to prescribed medication and lifestyle habits.	Usual care	1 year
Capomolla et al. (2002) [59]	RCT	112	122	234	HF	To compare the effectiveness and cost/utility ratio between a heart failure (HF) management program delivered by day-hospital (DH) and usual care in chronic heart failure.	A tailor-made treatment plan and interventions were structured for each patient, which included: cardiovascular risk stratification, individualised therapy according to guidelines, control of clinical stability according to EBM criteria, correction of risk factors, health education and counselling. The HF	Usual care	3 months

							management programme staff consisted of a cardiologist, nurses, physiotherapists, a dietician, a psychologist, and a social worker.		
Cavusoglu et al. (2017). [60]	RCT	125	123	248	HF II–IV LVEF <40%	To assess the efficacy and feasibility of an enhanced heart failure (HF) education with a 6-month telephone follow- up program in post-discharge ambulatory HF patients.	In the EHFP (Enhanced HF education follow-up programme) group, patients were instructed by a cardiologist and a nurse on the management of HF during discharge and were provided with an educational leaflet on HF and a home scale. Patients were contacted by telephone. During the telephone follow-up, correction of doses or the drug regimen was performed by a cardiologist or a nurse under the supervision of a cardiologist. Before starting the study, two cardiologists and two nurses from each centre participated in a training course on educating patients with HF.	Usual care	6 months
Chaudhry et al. (2010) [61]	RCT	826	827	1653	HF	To determine whether telemonitoring would reduce the combined end point of readmission or death from any cause among patients recently hospitalized for heart failure.	The telemonitoring group was instructed to make daily calls to the system's toll-free number. During each call, patients listened to a series of questions about their general health status and heart failure symptoms and entered their answers using the telephone keypad. Information from the telemonitoring system was downloaded daily to a secure Internet site and reviewed by the site coordinators.	Usual care	180 days
Chen et al. (2018) [62]	RCT	31	31	62	HF II- IV	To determine if a multidisciplinary disease management program (MDMP) improves quality of life (QoL), physical performance, depressive symptoms, self-care behaviours and mortality or rehospitalization in patients with HF in China.	The patients in the MDMP group were followed by the HF team consisting of 3 cardiologists, 1 nurse coach, 10 nurses, 1 dietician and 1 psychiatrist, and received an intensive multidisciplinary intervention in addition to standard care. The intervention included improved discharge education, exercise training and follow-up contacts. Patients who screened positive for depression were treated by a psychiatrist. Patients received a home visit by the nurse coach 2 weeks after discharge, telephone	Usual care	6 months

							contacts every 2 weeks and enhanced education at 90 days and 180 days.		
Clark et al. (2015). [63]	RCT	25	25	50	HF I-III	To examine the effects of an education-support intervention delivered in the home setting, using strategies to improve health status and self-care in adults/older adults with class I-III HF. The secondary purpose was to explore participants' subjective perceptions of the intervention.	The intervention consisted of an educational and skill-building programme with supportive telephone follow-up, delivered individually by APRNs specialised in clinical nursing. Strategies for self-efficacy included social persuasion and encouragement, targeted feedback, breaking down information into realistic segments, skill mastery and memory enhancement for healthy elderly. The first phase of the intervention included home visits, The second phase took place via telephone and/or e-mail with the APRN, without home visits.	Usual care	9 months
Cleland et al. (2005) [64]	RCT	NTS: 173<br>HT: 168	85	426	HF LVEF <40%	To identify whether home telemonitoring (HTM) improves outcomes compared with nurse telephone support (NTS) and usual care (UC) for patients with heart failure who are at high risk of hospitalization or death.	Patients in the intervention groups were randomly assigned to receive either the HTM or the NTS. Those in the NTS group were contacted by telephone every month by a heart failure nurse to assess their symptoms and drug therapy. Patients in the HTM group used telemonitoring equipment installed at home, which consisted of an electronic scale, an automatic sphygmomanometer, and an electrocardiograph, in addition to telephone assistance. Patients were asked to take a series of measurements which were transmitted automatically. Higher or altered values were automatically notified to the practice nurses, who reviewed the information and acted.	Usual care	240 days
Cline et al. (1998) [65]	RCT	80	110	206	HF	To study the effects of a management programme on hospitalisation and health care costs one year after admission for heart failure.	The patients and their families received an education programme on heart failure and guidelines for the self-management of diuretics according to the signs and symptoms of worsening heart failure. The educational programme consisted of two visits by a nurse	Usual care	1 year

							during the primary admission and a one-hour information visit for patients and relatives two weeks after discharge. The patients were cared for in an easily accessible outpatient clinic led by a nurse. The nurse was available by telephone during office hours.		
Crossley et al. (2011) [66]	RCT	1014	983	1997	HF	To determine if wireless remote monitoring with automatic clinician alerts reduces the time from a clinical event to a clinical decision in response to arrhythmias, cardiovascular (CV) disease progression, and device issues compared to patients receiving standard in-office care. A secondary objective was to compare the rates of CV health care utilization between patients in the remote and in-office arms.	After insertion of an ICD or CRT-D, patients were randomly assigned to either wireless remote monitoring or in-office care. All automatic physician alerts were activated for remote arm patients. Patients in the remote arm received a home monitor, and their in-office visits at 3, 6, 9, and 12 months were replaced with remote visits, including a remote device transmission. Clinicians had access to the entire set of device-collected diagnostics for all study patients.	Usual care	15 months
Cruz et al. (2010) [67]	RCT	275	137	412	HF I-IV	Testing the hypothesis that a continuous and long-term follow-up programme directed at patients with HF, implemented by a multidisciplinary team, based on phone-call monitoring and repetitive educational activities could produce different effects on each of the quality-of-life components.	The intervention included the education of patients and caregivers; communication was made during individual or group face-to-face interviews, as well as by telephone. Patients had their medical treatment optimized based on guidelines and received remote monitoring. The multidisciplinary team of care providers included nurses, cardiologists, pharmacists, social workers, dieticians, dentists, and psychologists.	Usual care	6 and 12 months and then annually up to 72 months.
Dansky et al. (2008) [68]	RCT	Not stated	Not stated	284	HF	To investigate the influence of telecare on self-management of heart failure in a sample of older adults.	The intervention (telehealth) group received standard home care, enhanced with a telehealth system placed by the nurse in the patient's home during the home visit, designed to operate over a standard telephone system, connecting a central station. Units included a monitor with a data port for peripheral devices that allowed	Usual care	60 and 120 days

							patients to take their own measurements and transmit the readings. Two kinds of systems were in use: video systems that promoted live interaction between nurse and patient, and monitoring systems that were initiated by the patient. Nurses and patients acted on abnormal readings as needed.		
Dansky et al. (2009) [69]	RCT	64	44	108	HF	To determine whether using a telehealth system after discharge from formal home health services would improve clinical outcomes and self-management behaviours.	The patients received the health Buddy and used it throughout the duration of the home healthcare services, which were scheduled to ask the patients HF-related questions. During the formal care phase, all patients received standard care by a home care nurse. Patients in the telecare group continued to use the health Buddy for 180 days after discharge from formal home health services; patients in the control group received no further telecare or home health services.	Usual care	3 months
Dar et al. (2009) [70]	RCT	91	91	182	HF II–IV	To examine the impact of home telemonitoring on typical heart failure patients discharged from three acute hospitals.	The patients in the intervention group received telemonitoring equipment installed at home. The equipment included an electronic scale, an automatic blood pressure cuff, a pulse oximeter and a control unit connected to the telephone line. The transmitted data were reviewed daily by a specialist nurse. Any variation of vital signs from the predefined parameters triggered an alarm and led to a telephone call.	Usual care	6 months
Davis et al. (2012) [71]	RCT	63	62	125	HF	To evaluate the effectiveness of a tailored educational intervention designed specifically for patients with MCI on heart failure knowledge, self-care behaviour, and readmission rates.	The intervention focused on manipulating the environment and training compensatory strategies to manage memory and executive functioning disorders and to improve self-confidence. During hospitalisation, patients also participated in individualised verbal training and interactive problem-solving sessions. The sessions were recorded on audiocassette; patients received a cassette player and a recording of their sessions and were encouraged to review it during their	Usual care	1 year

							hospital stay and after discharge. Patients received a follow-up phone call 24-72 hours after discharge to conduct a learning session.		
de la Porte et al. (2007) [72]	RCT	118	122	240	HF III-IV	To determine whether an intensive intervention at a heart failure (HF) clinic by a combination of a clinician and a cardiovascular nurse, both trained in HF, reduces the incidence of hospitalisation for worsening HF and/or all-cause mortality (primary end point) and improves functional status (including left ventricular ejection fraction, New York Heart Association (NYHA) class and quality of life) in patients with NYHA class III or IV.	The intervention consisted of an intensive follow-up of the patients during 1 year at an HF outpatient clinic led by an HF physician and a cardiovascular nurse. Comprehensive verbal and written education were given on the disease and aetiology, medication, compliance, and possible adverse events. Patients were informed about diet, weight control, early recognition of worsening HF, when to call a healthcare provider, exercise, and rest. A patient diary was provided. Easy access to the outpatient clinic during working hours was offered. An appointment with a dietician was arranged.	Usual care	12 months
de Souza et al. (2014) [73]	RCT	120	129	149	HF II–III LVEF ≤45%	To evaluate the clinical efficacy of a nurse-based strategy involving HVs and telephone reinforcement after ADHF admissions, compared with the conventional strategy primarily based on medical follow-up.	After hospital discharge, patients were randomly allocated to receive specialist nurse-led home visits and telephone calls or usual care (return to the outpatient clinic) over 6 months. Visiting nurses provided guidance on disease awareness, self-care practices, early recognition of the signs and symptoms of decompensation and decision making in the event of decompensation, annual immunizations, and the importance of adherence to a low-sodium diet, fluid restriction, and medications.	Usual care	6 months
DeBusk et al. (2004) [74]	RCT	234	228	462	HF	To determine whether a telephone-mediated nurse care management program for heart failure reduced the rate of rehospitalization for heart failure and for all causes over a 1 year period.	The standardised, telephone-mediated intervention comprised the following elements: initial educational session, including a videotape; basic telephone counselling session; nurse-initiated follow-up telephone contacts; pharmacological management; nurse-initiated communication with physicians. The nurse managers obtained permission from the physicians to initiate and adjust the	Usual care	1 year

							drug therapy for heart failure according to the study protocol.		
Del Sindaco et al. (2007) [75]	RCT	86	87	175	HF II-IV	To determine the long-term efficacy of a hybrid disease management programme (DMP) in consecutive older outpatients.	The intervention consisted of combined inpatient (cardiologists and nurse coordinators of two heart failure clinics) and home care (visits by the patient's general practitioner). The components of the DMP were the following: discharge planning, education, optimisation of therapy, improvement of communication, early attention to signs and symptoms. Intensive follow-up was based on scheduled hospital visits (starting 14 days after discharge), nurse's calls and GP visits at home.	Usual care	2 years
Dendale et al. (2012) [76]	RCT	80	80	160	HF LVEF 35	To test whether intensive follow-up of patients through a telemonitoring-facilitated collaboration between general practitioners (GPs) and a heart failure clinic could reduce mortality and rehospitalization rate.	Patients assigned to the intervention group received six months of intensive follow-up facilitated by telemonitoring (TM). On the day of hospital discharge, they were instructed how to use an electronic scale and monitoring device connected via Bluetooth to a mobile phone, which automatically forwarded the results to a central computer. The general practitioner and the heart failure clinic were alerted via an automatic email when measurements exceeded predefined alert levels. When this occurred, the general practitioner examined the patient. The nurse contacted the patient by telephone 1-3 days after the alert; there was no routine telephone contact.	Usual care	6 months
DeWalt et al. (2006) [77]	RCT	59	64	123	HF II-IV LVEF <40%	Comparing the efficacy of a heart failure self-management program designed for patients with low literacy versus usual care.	The intervention started with an educational session with a clinical pharmacist or health educator during a clinical visit. Patients were given an educational booklet designed for patients with poor reading skills. The health educator used pedagogical strategies deemed to improve comprehension for patients with poor literacy. The	Usual care	3 months

							programme coordinator then made scheduled follow-up phone calls.		
DeWalt et al. (2012) [78]	RCT	303	302	605	HF II-IV	To compare the effects of 2 different amounts of self-care training on the incidence of all-cause hospitalization and death and the incidence of HF-related hospitalizations and HFQOL.	All participants received the initial training session from trained bilingual health educators. After the initial training session, participants were randomised to either the single-session or the multi-session group. Those assigned to the multi-session intervention received the same initial session plus a more intensive self-care education and training intervention, which also included instruction on weighing oneself daily to guide diuretic self-regulation if the patient's doctor allowed it. In the following 4 weeks, patients in the multi-session group received follow-up telephone calls from the health educator.	Usual care	12 months
Domingo et al. (2012) [79]	Before-after	46	51	97	HF II–IV	To assess a telemedicine program in a HF Unit.	Motiva System + self-monitoring tools (scales and sphygmomanometer to take measurements every morning).	Motiva system only (educational videos, motivational messages, and questionnaires)	6 months 1 year
Domingues et al. (2011) [80]	RCT	57	63	120	HF LVEF ≤ 45%	To compare two nursing intervention groups among patients hospitalized due to decompensated HF. Outcomes were levels of HF and self-care knowledge, the frequency of visits to the emergency room, rehospitalizations and deaths in a three-month period.	Patients received nursing educational intervention during their hospitalisation, which provided comprehensive information on HF, pharmacological and non-pharmacological treatment. After discharge from hospital, patients in the intervention group were randomised to receive systematic telephone contact from the study nurse with the aim of clarifying and reinforcing the instructions received during admission, monitoring signs and symptoms of decompensation and investigating possible emergency room visits and re-hospitalisations.	Usual care	3 months

Doughty et al. (2002) [81]	RCT	100	97	197	HF	To determine the effect of an integrated heart failure management programme, involving patient and family, primary and secondary care, on quality of life and death or hospital readmissions in patients with chronic heart failure.	Individual education with the study nurse was started at the first clinical visit. The follow-up included 6-week visits alternating between the general practitioner and the outpatient clinic. Patients were offered group educational sessions on the management of HF, run by a cardiologist and the study nurse.	Usual care	1 year
Ducharme et al. (2005) [82]	RCT	115	115	230	HF	To determine the impact of care at a multidisciplinary specialized outpatient congestive heart failure clinic compared with standard care.	Individual education of the patient, family members or both with the practice nurse was initiated at the first clinical visit and reinforced at each subsequent visit.	Usual care	6 months
Duffy et al. (2010) [83]	RCT	15	17	32	HF III-IV	To describe the patient population, evaluate the result of the intervention on intended patient outcome, examine the resources required to implement the intervention, including nursing capacity and identify unanticipated problems or operational difficulties.	Intervention patients were assigned to 1 of 7 intervention nurses trained by the researcher in the study protocol. Nurses phoned patients and completed the telephone log after each telephone "visit" indicating the date and total time of the interaction; major focus of the intervention were symptom monitoring, education, and emotional support; patient's participation and response to the intervention; and nurse's opinion of the conduct and effectiveness of the visit.	Usual care	2 months
Dunbar et al. (2015) [84]	RCT	70	64	134	HF II–IV and type II DM	To test an integrated self-care intervention on outcomes of HF and diabetes mellitus (DM) QOL, physical function and physical activity (PA).	Participants in the intervention group participated in a personalised educational and counselling session. Motivational messages focused on the role of HF-DM patients in self-care, self-monitoring of glucose, weight and symptoms. At 48-72 hours, the nurse made a home visit to review glucose and weight information and the participant's interpretation of the data, followed by follow-up telephone calls.	Usual care	6 months
Ekman et al. (1998) [85]	RCT	79	79	158	HF III–IV	To evaluate the feasibility of a nurse-monitored, outpatient-care program for elderly patients previously hospitalized with chronic heart failure	The structured care programme was based on an outpatient clinic staffed by nurses, run in cooperation with the practice physicians, who were responsible for optimal drug treatment. Patients were instructed to call the nurse in the event of worsening symptoms or disease-related questions. An attending	Usual care	6 months

							physician was responsible for medical decisions. In addition to the outpatient visits, the nurse regularly contacted the patients by telephone.		
Eyre et al. (2016) [86]	RCT	25	25	50	HF LVEF ≥45%	The Rehabilitation EnAblement in CHronic Heart Failure in patients with Heart Failure (HF) with preserved ejection fraction (REACH-HFpEF) pilot trial is part of a research programme designed to develop and evaluate a facilitated, home-based, self-help rehabilitation intervention to improve self-care and quality of life (QoL) in heart failure patients and their caregivers.	The REACH-HF intervention is a comprehensive self-care support programme comprising the 'Heart Failure Manual' with a choice of two exercise programmes for patients, a 'Resource for Family and Friends' for caregivers, and a 'Progress Tracker' tool for patients. Patients and caregivers will work through the manual with the guidance of a specially trained cardiology nurse, who will help develop an understanding of how to manage HF. Patients will be advised to use the Progress Tracker, designed to encourage patients to monitor their condition and make associations between improvements in self-care and improvements in symptoms/well-being. The Family and Friends Resource, a manual for use by caregivers, contains advice on how to provide support to the person with heart failure.	Usual care	6 months
Feldman et al. (2005) [87]	RCT	339	448	1242	HF	To assess the impact and cost-effectiveness of two information-based provider reminder interventions designed to improve self-care management and outcomes of heart failure (HF) patients.	Nurses were assigned to either a control group (usual care) or one of two intervention groups (basic or augmented). Both basic and augmented interventions provided the nurse with an e-mail reminder highlighting six HF-specific clinical guidelines. In addition, nurses in the augmented group received a laminated card focused on medication management, a prompter card to facilitate better physician–nurse communication, a self-care guide for patients, and follow-up outreach by a clinical nurse specialist.	Usual care	45 days

Feldman et al. (2004) [88]	RCT	276	254	530	HF	Examining the effects of a home health intervention designed to standardize nursing care, strengthen nurses’ support for patient self-management and yield better CHF patient outcomes.	The intervention consisted of: (1) an evidence-based nursing protocol that adapted the heart failure guideline; (2) a patient self-care guide published in English and Spanish with illustrations and language accessible to someone with a sixth grade education; (3) an interactive practitioner training utilized experienced facilitators, as well as role-playing and audiotaping, to help nurses increase their skills in communicating with and motivating their patients to adhere to treatment instructions.	Usual care	3 months
Ferrante et al. (2010) [89]	RCT	760	758	1518	HF	Assess the rate of death and hospitalization for heart failure (HF) 1 and 3 years after a randomized trial of telephone intervention aimed to improve education and compliance in stable patients with HF ended.	Patients received an explanatory leaflet and were followed up with a telephone intervention by trained nurses. The objectives of the intervention were to improve diet and treatment adherence, promote exercise, regularly monitor symptoms, weight, and oedema, and promote timely visits if signs of clinical deterioration were detected. Patients were initially called every 14 days, and, after the fourth call, the frequency could be adjusted according to the severity of each case and patient compliance. More severe and less compliant patients received more calls.	Usual care	3 years

Finkelstein et al. (2010) [90]	Mixed method study	NA	NA	10	HF	To design a low cost tele management system for CHF patients and to perform an initial assessment of patient acceptance of such a system.	The Home Automated Tele management (HAT) system for HF comprises a home unit (computer and electronic scale), a decision support server and a web-based clinical portal. Patients answer questions regarding symptoms, side effects, adherence and receive specific education using the home unit. Results are transmitted to the decision support server after each self-test session. The care management team customises alerts and action plans for each patient. The action plan defines the self-management actions to be taken by the patient based on the self-test data and can be displayed on the HAT home unit and on the physician's website.	**NA**	1 year
Flynn et al. (2005) [91]	Before after study	NA	NA	31	HF I-III LVEF ≤40%	To examine whether a year-long group-based self-management intervention is feasible.	The intervention comprised a total of 18 90-minute sessions using the principles of social cognitive theory offered over the course of one year.	NA	52 weeks
Galbreath et al. (2004) [92]	RCT	710	359	1069	HF I-IV	To evaluate the effectiveness of disease management (DM) as a clinical and cost-containment strategy in both systolic and diastolic CHF over a longer time and with a larger and more heterogeneous sample than those of previous studies.	All DM patients received bathroom scales. Within the treatment group, there was additional randomization, with half of the treatment group given in-home technology, including an electronic blood pressure monitor and finger pulse oximeter. Blood pressure and pulse oximeter readings were reported by the patient to the disease managers. This group also wore activity monitors for 2 of each 6 weeks and had thoracic bioimpedance cardiac output measurements performed at 6-month intervals. The disease manager, a registered nurse with specialised cardiac training, provided patient education and medication management in conjunction with the PCP. The DM program was administered telephonically.	Usual care	18 months

Gattis et al. (1999) [93]	RCT	90	91	181	HF LVEF <45%	To evaluate the effect of a clinical pharmacist on outcomes in outpatients with heart failure. The primary end point was the composite of all-cause mortality and nonfatal heart failure events. Use and dose of ACE inhibitors prescribed were evaluated as secondary objectives.	The clinical pharmacist discussed the patient's case and verbally provided therapeutic recommendations on optimising therapy to the treating physician. The focus was on optimising heart failure therapy, avoiding digoxin toxicity, avoiding contraindicated drugs or drug interactions. Patients had the opportunity to ask questions to the pharmacist and were given a telephone number to contact him. The clinical pharmacist performed a telephone follow-up. Patient assessment and education were provided by the attending physician and/or physician's assistant or nurse.	Usual care	24 months
Giordano et al. (2009) [94]	RCT	230	230	460	HF LVEF <40%	To determine whether a home-based tele management (HBT) programme in CHF patients decreased hospital readmissions and hospital costs in comparison with the usual care (UC) follow-up programme over a one-year period.	HTB is a multidisciplinary care approach of medical and nursing interventions performed by telephone, with the possibility of transmitting an ECG trace to a workstation. Patients received, prior to hospital discharge, a hand-held device that transferred, via a landline or mobile phone, a trace to a receiving station, where a nurse or doctor was available 24 hours a day, 7 days a week. Medical and nursing interventions were carried out by telephone. During scheduled telemonitoring, the nurse carried out educational interventions; occasional telecare appointments were made when the patient, in the presence of symptoms or signs of decompensation, called the nurse.	Usual care	1 year
Goldberg et al. (2003) [95]	RCT	138	142	280	HF III- IV LVEF 35%	To determine whether a technology-based, physician-directed daily weight and symptom monitoring system would reduce rehospitalization rates; to assess the impact of the technology on mortality and health-related quality of life in patients admitted to the hospital with decompensated advanced heart failure secondary to systolic dysfunction.	Patients randomised to the intervention received a monitoring system with home monitors. The system comprised an electronic scale and an individualised symptom response system (monitor) connected to a computerised database monitored by nurses. Patients were instructed to weigh themselves and answer yes/no questions about heart failure-related symptoms twice a day. The attending physician identified symptom questions and weight goals for each patient at the time of	Usual care	6 months

							enrolment. The nursing staff examined the patients' weights and responses daily and contacted the patient if necessary. Weight increases beyond a predetermined amount and/or changes in the patient's symptoms were promptly reported to the physician by the nurses.		
Gonzalez et al. (2005) [96]	Prospective cohort study	NA	NA	298	HF III-IV	To evaluate what is really achieved with nurse education in an outpatient heart failure population.	To find out patients' compliance, their level of knowledge of the disease and treatment, and their level of self-care, a questionnaire was completed by the nurse. Cardiac performance and knowledge and understanding of HF were classified into four categories, using the nurse's subjective estimation. Treatment compliance was assessed using a personal interview.	NA	1 year
Gonzalez-Guerrero et al. (2015) [97]	RCT	59	57	116	HF	To compare the effectiveness of a disease management programs (DMP) with usual care in elderly adults with HF examined whether the intervention was effective in increasing event free survivals (defined as the time elapsed until the first readmission or until death of the individual from any cause during the study period) in individuals with cognitive impairment.	The intervention group received the DMP, consisting of comprehensive hospital discharge planning and close follow-up at a geriatric day hospital (GDH) by a multidisciplinary team consisting of a geriatrician (case manager), a nurse and a social worker after hospital discharge. The participants were monitored via telephone contacts and in-person visits at the GDH. During telephone contacts, the team provided educational reinforcement and assessed possible heart failure	Usual care	12 months
Gotsman et al. (2011) [98]	Prospective cohort study	NA	NA	324	HF II-IV	To evaluate the clinical outcome of patients with HF treated at a multidisciplinary HF Center.	A multidisciplinary team comprising a HF nurse, a cardiologist specialized in HF management and a dietician evaluated patients admitted to the HF center. Patients received detailed information about their disease from the cardiologist and the HF nurse. The nurse followed the patients regularly by telephone and/or by periodically scheduled clinic visits depending on the clinical status of the patient. In addition, regular follow-up clinic visits with the HF specialist were	NA	24 months

							scheduled, depending on the patients' clinical status.		
Granger et al. (2009) [99]	Qualitative	NA	NA	12	HF	To examine how communication about the HF regimen between patients and their physicians is experienced and understood by both partners.	Patients and physicians were interviewed individually using open-ended research questions to guide the interview. Patients' self-care and ability to manage a chronic illness regimen were explored. How communication and information exchange about the prescribed HF regimen was experienced and understood by both patients and physicians was investigated.	NA	NA
Hanchett et al. (1967) [100]	RCT	126	113	239	HF	Determine if the hospital admission rate of patients with chronic congestive heart failure could be reduced by adding public health nursing follow up at home to the regular routine of outpatient department care.	The patients in the study group received public health nursing follow-up. Each patient received a home visit from one of the nurses. During this home visit, the nurse took note of the patient's living conditions and tried to assess the patient's understanding of his illness and his adherence to the treatment plan. A nurse was present in the clinic at the time of the patient's visit to facilitate the transfer of information with the doctor. In between clinic visits, nurses visited patients at home, either on their own initiative, on doctor's instructions or at the patient's request.	Usual care	Flexible plan
Harrison et al. (2002) [101]	RCT	192	100	92	HF	To evaluate whether the use of usual providers, and a reorganization of discharge planning and transition care with improved intersect or linkages between nurses, could improve quality of life and health services utilization for individuals admitted to hospital with heart failure.	Patients in the transitional care (TC) arm received discharge planning and standard care, as well as a comprehensive programme that added support to enhance the transfer from hospital to home. To develop this programme, hospital and community nurses met to identify gaps and the 3 main aspects of the transition from hospital to home: supportive care for self-management; linkages between nurses and patients in the hospital and home; and the balance of care between the patient, family, and professionals. The TC is based on the use of a structured, comprehensive, and evidence-based protocol for counselling and	Usual care	3 months

							education on self-management of heart failure.		
Hebert et al. (2008) [102]	RCT	203	203	406	HF	To estimate the cost-effectiveness of a nurse-led disease management intervention over 12 months, implemented in a randomized, controlled effectiveness trial.	Nurse-led program in which patients had 1 in-person visit with a trained nurse and periodic follow-up telephone calls. The nurses stressed adherence to a low-salt diet and to medications and worked with the patient's physician to optimise heart failure medications according to published guidelines.	Usual care	1 year
Heisler et al. (2007) [103]	Before-after	NA	NA	20	HF II-III	To evaluate the feasibility and acceptability of an interactive voice response (IVR)–based platform to facilitate telephone peer support among older adults with HF	During the face-to-face session, participants received instructions and written material on how to use the IVR system; they also participated in an interactive training session on peer communication techniques based on motivational interviewing, developed, and conducted by an expert, to introduce key communication techniques. Participants were asked to contact the partner at least once a week using the free IVR telephone line.	**NA**	7 weeks
Hershberger et al. (2001) [104]	Before-after	NA	NA	108	HF	To evaluate the clinical and cost outcomes of care provided by this heart failure outpatient management program and to show the structure and process of care that have been used during the past several years.	The outpatient heart failure management programme consisted of cardiologists, nurses, and a social worker. Patients were seen in a collaborative manner. After the initial admission visit to the clinic, the nursing coordinator contacted the patients weekly or biweekly. When symptoms, physical signs or laboratory abnormalities appeared, the patient was reviewed by the medical staff and interventions were undertaken. Patient education was reinforced during each visit by both physicians and nursing coordinators with reminders of the educational intervention.	NA	6 months

Holland et al. (2007) [105]	RCT	149	144	293	HF	To test whether a drug review and symptom self-management and lifestyle advice intervention by community pharmacists could reduce hospital admissions or mortality in heart failure patients.	The pharmacists educated the patient/caregiver about heart failure and their medication and provided basic advice on exercise, diet and smoking cessation. They also encouraged the completion of simple sign and symptom monitoring forms, provided recommendations to the general practitioner, and informed the local pharmacist of the possible need for medication adherence aids.	Usual care	8 weeks
Hui et al. (2006) [106]	Before after	NA	NA	37	HF	To develop and evaluate a community-based programme for heart failure patients, with emphasis on exercise and education.	12-week programme consisted of didactic lectures on anatomy and physiology, diet, monitoring of signs and symptoms, and exercise. Exercise training was included: warm-up, upper and lower limb strengthening with the use of graduated elastic bands, and aerobic dance.	NA	3 months
Inglis et al. (2006) [107]	RCT	149	148	297	HF II-IV LVEF 55%	To examine the long-term (minimum of 7.5 to 10 years) impact of a nurse-led, multidisciplinary home-based intervention (HBI) relative to usual post discharge care (UC) in a typically old and fragile cohort of CHF patients initially discharged from short-term hospital care.	The HBI included a structured home visit within 7-14 days after discharge by a nurse and a pharmacist or a qualified cardiologist nurse. During the home visit, patients underwent a physical examination and a review of adherence and knowledge of their condition and prescribed treatments, as well as an assessment of their social support system. Subjects with signs of clinical deterioration were immediately reviewed by the general practitioner or cardiologist and corrective measures were taken. All patients underwent telephone follow-up.	Usual care	10 years
Jaarsma et al. (1999) [108]	RCT	84	95	179	HF III-IV	To test the effect of education and support by a nurse on self-care and resource utilization in patients with heart failure.	The supportive educational intervention consisted of intensive education by a study nurse on the consequences of heart failure. During the hospital stay, the nurse assessed the patient's needs, provided education and support, and gave the patient a card with warning symptoms. Within a week of discharge, the practice nurse telephoned the patient to assess potential problems and make an appointment for a home visit. During the home visit, the nurse	Usual care	9 months

							reinforced the instruction. Between discharge and the home visit, patients could call the nurse if they had problems.		
Jaarsmaet al. (2000) [109]	RCT	55	73	128	HF I-IV	Describe heart failure-related self-care behaviour, to test the effect of education and support on self-care behaviour and to discuss limitations.	Structured patient education, a follow-up telephone call or a home visit from a nurse.	Usual care	1,3 and 9 months
Jaarsma et al. (2000) [110]	RCT	58	74	186	HF III-IV	To determine the effects of a supportive educational nursing intervention on self-care abilities, self-care behaviour, and quality of life of patients with advanced heart failure.	During the hospital admission, the study nurse assessed the patients' educational and counselling needs, provided education and support to the patient, gave the patient a card with alarm symptoms and discussed discharge issues. Within one week after discharge, the nurse called the patient at home to assess potential problems and make an appointment for a home visit. During the home visit, the practice nurse reinforced and continued the education.	Usual care	9 months
Jaarsma et al. (2004) [111]	RCT	350	350	1050	HF II–IV	To determine the effectiveness of two interventions: compared to ‘care as usual’ in HF patients, on time to first major event (HF readmission or death), quality of life and costs. To investigate the role of underlying mechanisms (knowledge, beliefs, self-care behaviour, compliance) on the effectiveness of the two interventions.	Patients in intervention group 1 will be provided with education in the hospital and during outpatient visits and behavioural strategies to improve compliance. A telephone contact will be made within 2 weeks after discharge. They will have telephone access to a specialist nurse. Patients in intervention group 2 will receive intensive counselling and support. In addition to the interventions in intervention group 1 they will receive a home visit from the nurse, they will be contacted every month by the nurse. The nurse will consult a multidisciplinary team consisting of a physiotherapist, dietician, and social worker.	Usual care	1, 6, 12, and 18 months
Jaarsma et al. (2008) [112]	RCT	348	348	1049	HF II-IV	To examine the effects of a nurse-led disease management program in a sufficiently large population with an assumed relatively high	Patients in the basic support group were visited by the HF nurse at the outpatient clinic and behavioural strategies were used to improve adherence. Patients in the intensive support group additionally received	Usual care	18 months

						event rate. To determine the effects of 2 levels of intensity in disease management programs on the combined end points of death and readmission to the hospital.	monthly contact with the nurse and multidisciplinary counselling provided by a physiotherapist, a dietician, and a social worker. The materials used included a patient diary, brochures on HF and samples of reduced-sodium food condiments.		
Jerant et al. (2003) [113]	RCT	12	12	37	HF	To clarify the question of whether home telecare "adds value" to standard telephone monitoring by comparing the effectiveness of three post-hospital discharge care models for reducing congestive heart failure (CHF) related hospital readmission charges.	Patients assigned to telephone care received scheduled telephone calls from the study nurse, while those assigned to the video telecare group received scheduled telecare visits at home using a device that enabled real-time video conferencing with the nurse; it included a microphone, an electronic stethoscope and a small camera that allowed observation of facial expressions, respiratory effort, lower limb oedema and scale displays.	Usual care	6 months
Jerant et al. (2005) [114]	Qualitative	NA	NA	54	Chronic conditions	To elicit perceived barriers to active self-management and to accessing self-management support resources of chronic conditions.	The moderator (MVF) asked each focus group participant to state their chronic conditions and to note how they impacted on their lives. The moderator then presented a series of pre-written questions. Methods used to 'flush out' quiet subjects and facilitate detailed responses included the use of silence, non-threatening body posture and eye contact, and open-ended responses.	NA	**NA**
Karlsson et al. (2005) [115]	RCT	103	105	208	HF II-IV LVEF < 45%	To assess the effect of a nurse-based management program aimed to increase heart failure patients' knowledge about disease and self-care and to relate the results to gender and cognitive function.	The nurse-monitored management programme for patients with heart failure is supervised by a senior cardiologist. The patient comes to the outpatient clinic and is encouraged to maintain contact with the nurse. At each visit, the nurse checks for symptoms and signs of heart failure, blood pressure, heart rate and weight, and laboratory tests are performed. The nurses are authorised to set and change the doses of angiotensin-converting enzyme (ACE) inhibitors, angiotensin II receptor blockers, β-adrenoceptor blockers, potassium, and diuretics according to a standard protocol. The patient is instructed to self-monitor and in the presence of good compliance to	Usual care	6 months

							change the dosage of diuretics autonomously.		
Kasper et al. (2002) [116]	RCT	102	98	200	HF III-IV LVEF < 35%	To determine whether a multidisciplinary outpatient management program decreases chronic heart failure (CHF) hospital readmissions and mortality over a six-month period.	The telephone nurse coordinator made follow-up calls to the patients and addressed the problems according to clinical indications but did not change the medication. The nurses contributed to the implementation of the treatment plan designed by the cardiologists and modified the medication according to the cardiologists' instructions. GPs managed non-HF-related problems, received updates from nurses and were informed of abnormal laboratory values. All patients in the intervention group were provided with a pill sorter, a list of correct medication, a list of diet and exercise recommendations and educational material.	Usual care	6 months
Khunti et al. (2007) [117]	RCT	1686	1345	3031	HF	To evaluate the effect of a disease management programme for patients with coronary heart disease (CHD) and chronic heart failure (CHF) in primary care.	In the intervention group, two peripatetic nurse specialists trained in the management of CHD and CHF travelled between practices, where they held weekly clinics. The nurse intervention included patient assessment, confirmation of diagnosis by investigations, medication management and titration, home visits for housebound patients with CHF and liaison between primary and secondary care. The nurses had the facility to refer patients for echocardiography and for assessment in a secondary care cardiology clinic.	Usual care	12 months
Kimmelstiel et al. (2004) [118]	RCT	97	103	200	HF	To investigate the short- and long-term impacts of a 3-month nurse-driven HF DM program on HF hospital use in patients recently hospitalized for HF who were already well managed with appropriate medications.	The nurse-manager, an expert in HF management, conducted a home visit focusing on dietary and medical compliance, daily weights, self-monitoring, and timely reporting of changes in weight or clinical status. During this session, patients and family members received a pre-printed patient and	Usual care	90 days

							family handbook. The nurse performed a cardiovascular examination and symptom assessment. Depending on the clinical status, the nurse-manager telephoned the patients weekly or biweekly and the information discussed during the home visit was reinforced. The nurse-managers were available by telephone 24 hours a day, 7 days a week and communicated frequently with the GPs		
Kline et al. (2007) [119]	RCT	340	399	1023	HF II-IV	To determine the effects of 2 levels of intensity in disease management programs on the combined end points of death and readmission to the hospital.	Supportive-educative (SE) group received the self-care management strategies based on Orem conceptual model. The MGS' nursing approach was developed from the conceptualisation of mutuality and King's goal attainment theory, a process by which nurses and patients collaboratively identify patient goals and the means to achieve them.	Instruction about health promotion activities	18 months
Koehler et al. (2011) [120]	RCT	354	356	710	HF II or III LVEF 35%	To determine whether physician-led remote telemedical management (RTM) compared with usual care would result in reduced mortality in ambulatory patients with chronic heart failure (HF).	The RTM system is based on a wireless Bluetooth device, together with a personal digital assistant and data are transmitted using mobile phone technology. The sensor network included a 3-lead ECG, a blood pressure device and a scale. The telemedicine centres provided 24/7 medical assistance. The patient was contacted by the physician at the telemedicine centre to verify measurements, provide advice or institute treatment. The telemedicine centre contacted the patient's local doctor every 3 months.	Usual care	12 months
Koelling et al. (2005) [121]	RCT	107	116	223	HF LVEF <40%	To determine whether a patient discharge education program would improve clinical outcomes in patients with chronic heart failure.	The patient education programme included a 60-minute individual teaching session with a nurse educator on heart failure that covered the basics of the causes of heart failure, the rationale for pharmaceutical therapies, the role of dietary sodium restriction, and limiting water intake. In addition, the patient education session	Usual care	180 days

							contained the rationale for self-care behaviours.		
Kommuri et al. (2012) [122]	RCT	128	127	265	HF I-IV LVEF 40%	To examine the changes in performance on heart failure knowledge assessments administered before and after discharge education.	Usual care plus a 1-hour heart failure education programme given by a nurse educator. The intervention group also received guidelines for the management of heart failure written in simple terms.	Usual care	3 months
Krumholz et al. (2002) [123]	RCT	44	44	88	HF	To determine the effect of a targeted education and support intervention on the rate of readmission or death and hospital costs in patients with heart failure.	An experienced cardiology nurse educated the patients during a face-to-face debriefing session within two weeks after discharge from hospital. The intervention was based on five domains of care, including knowledge of the disease, the relationship between medication and disease, the relationship between health behaviour and disease, knowledge of the first signs and symptoms of decompensation, and where and when to obtain care. Subsequently, the nurse contacted the patient by telephone for a total period of one year.	Usual care	10 months
Kwok (2008) [124]	RCT	49	56	105	HF	To evaluate the effectiveness and cost-effectiveness of a community nurse-supported hospital discharge programme in preventing hospital re-admissions, improving functional status and handicap of older patients with chronic heart failure.	The subjects were visited by a community nurse (CN) before being discharged from hospital. The aim was to provide health advice, such as medication compliance, dietary advice and to encourage subjects to contact the CN via a hotline during office hours when they developed symptoms. Subjects were visited by the CN at home within seven days after discharge. The CN made home visits at weekly intervals for a further four weeks and then monthly. The CN was in close contact with a geriatrician or cardiologist from the respective hospitals. After the liaison, the CN could change the medication regimen, organise urgent outpatient hospital appointments and clinical admissions.	Usual care	6 months

LaFramboise et al. (2009) [125]	Qualitative	NA	NA	31	HF	To discover perception about ease of use, efficacy and difficulties encountered by patient who used an in-home telehealth communication device.	Data collection included use of both focus groups and individual interviews, all of which were conducted by one of the research nurses.	NA	6-24 months
Lainscak et al. (2004) [126]	Before-after	NA	NA	50	HF LVEF < 45%	To assess the efficacy of intensive patient management in heart failure clinics with respect to patient knowledge, pharmacological management, and quality of life.	Multidisciplinary approach to the management of heart failure consisted of 45-minute consultation with the physician, detailed interview with emphasis on medical history, a physical examination, and an educational intervention. Further 10-minute sessions by a nurse on lifestyle changes, danger signs of worsening or deterioration and self-management were part of the subsequent visits.	NA	298 days in average
Landolina et al. (2012) [127]	RCT	99	101	200	HF LVEF ≤35%	To determine whether remote monitoring was associated with a different rate of emergency department and urgent in-office visits for HF, arrhythmias, or ICD-related events from patients in the standard arm.	The Care Link system performs the complete interrogation and transmission of ICD data via a standard telephone connection; the system alerts the physician via telephone or e-mail. In the remote arm, all alerts related to clinical management (intrathoracic impedance for monitoring fluid accumulation, atrial arrhythmias, ICD shocks delivered) were activated for wireless notification; no audible alerts were used. The clinics checked the Care Link website at least once a day for transmissions.	Usual care	16 months
Lang et al. (2018) [128]	RCT	25+25	25	50	HF LVEF ≥45%	To assess the feasibility and acceptability of the novel Rehabilitation EnAblement in CHronic Heart Failure (REACH-HF) rehabilitation intervention for patients with heart failure with preserved ejection fraction (HFpEF) and their caregivers.	Participating patients and caregivers read the REACH-HF manual with the facilitation of two qualified cardiology nurses. The facilitators provided support as needed, at least one in person and two through telephone contact. The REACH-HF manual includes a walking programme or a chair exercise DVD, or a combination of the two, stress management, monitoring of HF symptoms, medication intake and self-management.	Usual care	3 and 6 months
Laramee et al. (2003)	RCT	141	146	287	HF I-IV	To test the effect of CHF case management (CM) on the 90-day readmission	The intervention managed by a case manager consisted of early discharge planning and care	Usual care	3 months

[129]						rate in a heterogeneous setting.	coordination; patient and family education; 12 weeks of telephone follow-up and surveillance; and promotion of HF medication and medication doses (ACEI or ARB and BB) based on guidelines. The CM assisted the patient in coordinating care, facilitating the discharge plan, and obtaining the necessary referrals from social services, dietary services and physical/occupational therapy.		
Ledwidge et al. (2003). [130]	RCT	47	51	98	HF IV	To examine the cost-benefits of Multidisciplinary care (MDC) of HF in the setting of optimal medical care.	Patients systematically received nurse-led education and dietary consultations on three or more occasions during their hospitalisation. The patient was contacted by telephone 3 days after discharge and every week thereafter for up to 12 weeks, except for weeks 2 and 6, when patients and their close relatives attended the clinic. HF The purpose of the telephone call was to reinforce educational points.	Usual care	3 months
Leventhal et al. (2011) [131]	RCT	22	20	42	HF	To test the effect on hospitalisation, mortality, and quality of life of an adult ambulatory disease management programme for patients with HF in Switzerland.	After discharge from hospitalisation or rehabilitation, patients received a home visit from an HF nurse, followed by telephone calls over the following 12 months. The home visit consisted of a physical, psychosocial and environmental assessment, provision of educational, behavioural and self-care support. A kit was delivered that included in-depth explanations on HF and self-care procedures. After the home visit, a personalised nursing care plan was drawn up that included the goals identified by the patient and those identified by the nurse based on the results of the assessments then discussed with the GP.	Usual care	12 months
Liljeroos et al. (2015). [132]	RCT	71	84	155	HF II-IV	To determine the 24-month effects of an intervention with psych-educational support in dyads of heart failure patients and their partners.	The intervention was delivered through face-to-face counselling with an experienced HF nurse who had received special training, a computer-based programme and written material. The sessions were conducted in the couple homes or in the heart failure clinic.	Usual care	24 months

Linné et al. (2006) [133]	RCT	122	108	130	HF I	To evaluate the impact of added CD-ROM education on readmission rate or death for 6 months.	Patients randomised to the intervention group received additional training through an interactive programme on heart failure, its symptoms and treatment. The programme lasted approximately 20-30 minutes. After discharge from usual care, the patients returned to the hospital after two weeks and the CD programme was repeated.	Usual care	6 months
Liu et al. (2012) [134]	RCT	53	53	159	HF LVEF < 40%	To test whether integration of Oedema Index-guided management improved the 6-month outcomes of HF patients under multidisciplinary care.	In the case management (CM) group, the patient was assisted by an HF team (cardiologists, psychologist, dietary assistant and case manager). The team provided education on HF and self-monitoring methods, optimised HF medication and provided further cardiac and laboratory evaluations. Management consisted of prearranged consultations with the cardiologist and case manager, telephone contact every month and 24/7 telephone access to a case manager. Patients in the and EI-led CM group (EI) group received the same case management programme. However, the HF team used the EI value, measured at scheduled visits.	Usual care	6 months
López Cabezas et al. (2006) [135]	RCT	70	64	134	HF Framingha m	To assess the efficacy of a multifactorial educational intervention carried out by a pharmacist in patients with HF.	The patients in the intervention group were included in the active information programme. On the day of discharge from hospital, a personal interview was conducted in simple language, supported by audio-visual, and written educational material, which covered information on the disease, nutrition education and information on drug therapy. Every month during the first 6 months of follow-up, and every 2 months thereafter, a telephone call was made to the patient's home.	Usual care	12 months
Lowery et al. (2012) [136]	Quasi-experi-mental study	457	510	969	HF LVEF ≤40%	To compare patient outcomes under a nurse practitioner-led disease management model (intervention group) with outcomes under usual care (control group) in both	Both the patients and the NPs were recipients of the intervention. The program included an initial training component for the NPs that covered clinical management of CHF patients; a weekly videoconference sessions were attended by all of the	Usual care	24 months

						primary and tertiary medical centres.	NPs, as well as the CHF cardiologist at the lead centre, and provided an opportunity for discussion of new cases. The NPs communicated to patients face-to-face during clinic visits and via telephone to follow-up on patients' health status, especially patients undergoing diuresis or medication titration.		
Lupon et al. (2008) [137]	Prospective cohort study	NA	NA	151	HF	To assess patients' self-care progress after a medium-term period of nurse education intervention.	Patients were visited by the nurse at least every 3 months and by doctors every 6 months, and as many times as needed for up-titration of drugs and as many times as needed for manage their clinical situation. At every visit the nurse checked patients' smoking, drinking, alimentary and exercise habits and patients' treatment knowledge and compliance, tried to reinforce self-care behaviour and reinstructed patients about the disease.	NA	1 year
Mao et al. (2015) [138]	RCT	174	175	349	HF II-IV	To determine the effectiveness of MDP for patients with acute or decompensated chronic heart failure.	The patients were followed by a heart failure team consisting of two cardiologists specialised in heart failure care, a psychologist, a dietary assistant, a pharmacist and two case manager nurses. The team provided personalised education. In order to increase the rate of medication utilisation, a system was set up that included: a pathway that reminded nurses and physicians of medications; a medication checklist owned by the case managers; a computer that required discharge orders to be banned if there was no reason to explain the non-use of the medication based on the guidelines; and a pharmacist who provided education to the patient on the correct use of the medication.	Usual care	2 years
McCauley et al. (2006) [139]	RCT	/	/	118	HF	To examine interventions used by advanced practice nurses (APNs) to improve patient or caregiver self-management skills, provider understanding of patient needs, and overall management of HF and comorbid conditions.	The APNs visited the patient daily during admission, collaborated with the healthcare team and the patient's family to ensure optimal discharge planning, and visited the patient at home within 24 hours of discharge, at least weekly for the first month and at least bimonthly for the following 2 months. Patients and	Usual care	52 weeks

							caregivers had telephone access to APNs between visits. The APNs had access to experts in HF, pharmacology, and nutrition. They discussed challenging cases in bi-monthly conferences attended by the expert in the field to be discussed.		
McDonald et al. (2002) [140]	RCT	51	47	98	HF	To determine whether multidisciplinary care (MDC) of heart failure (HF) can reduce readmissions when optimal medical care is applied in both intervention and control groups.	The patients were systematically educated by a specialist nurse and consulted with a specialist dietician during their hospitalisation. After discharge, the management of HF-related problems was left to the clinic or nurse and not to the referring physician. The same nurse contacted the patient by telephone every week for up to 12 weeks to ascertain the clinical status and discuss any problems. Patients went to the HF outpatient clinic for scheduled check-ups. Patients were also asked to contact the HF outpatient clinic in case of clinical deterioration.	Usual care	3 months
Mehralian et al. (2014) [141]	Before after	11	11	22	HF II–IV, LVEF 45%	To determine the effects of education provided by nurses on the QOL in patients with CHF in home-visit care compared to usual care.	The patients in the intervention group received special education about their illness from nurses who visited the patients at home. The education included: information about the disease, the usual signs and symptoms and potential complications of the disease, prescribed medication, possible lifestyle changes, and the signs and symptoms they need to know to get to hospital on time.	Usual care	6 months
Mejhert et al. (2004) [142]	RCT	103	105	208	HF II–IV	To evaluate the effects of a nurse-based outpatient management programme for elderly patients discharged with heart failure from a university hospital.	Nurses monitor a management programme for patients with heart failure. Patients visits the outpatient clinic regularly and at each visit the nurse checks the symptoms and signs of heart failure, blood pressure, heart rate and weight. The nurses working in the programme are authorised to institute and modify the doses of angiotensin-converting enzyme (ACE) inhibitors, angiotensin II receptor blockers, b-adrenoceptor blockers, potassium substitutes, diuretics, and	Usual care	6, 12, and 18 months

							potassium-sparing diuretics according to a standard protocol.		
Miche et al. (2009) [143]	Non-randomized study	46	70	116	HF LVEF < 45%	To record health-related quality of life (QOL) in elderly patients with CHF and to assess the efficacy of a training program, as well as to evaluate any changes detected during a six-month follow-up.	A special educational program was developed for patients with heart failure, taking place once a week in groups of up to 10 patients. Teaching covered: medication and side-effects, the pathophysiology of patient diseases, a particular focus on compliance, diet, and nutrition. Effort was especially made to inform patients about physical exercise training within the context of their clinical picture. Trained staff (physician, psychologist, dietary assistant, sports therapist) conducted the lessons and gave individual training.	NA	6 months
Moertl et al. (2013) [144]	RCT	96	90	278	HF III-IV LVEF <40%	To present a cost utility analysis using prospectively collected data of QoL, outcome, and cost estimates from two different healthcare systems.	The MC included care by a heart failure nurse, which included home visits and telephone contact, pre-programmed consultations by the heart failure specialist, and on-demand consultations that were performed if the heart failure nurse noticed a deterioration in the patient's condition. Each consultation consisted of a physical examination and a 12-lead electrocardiogram was examined; laboratory tests were performed. In patients with an NT-proBNP level at discharge 2,200 pg/ml (BM), outpatient visits to a specialist were made at least every 2 weeks, in addition to the MC, to optimise medical therapy as quickly as possible	Usual care	18 months
Mortara et al. (2009) [145]	RCT	301	160	461	HF II-IV LVEF ≤40%	To assess the feasibility of a new system of home telemonitoring (HT). The HT system was used to monitor clinical and physiological parameters, and its effectiveness (compared with usual care) in reducing cardiac events	Patients assigned to HT were randomised into three groups. The first group (strategy 1) received monthly supportive telephone contact from a study nurse. The second group (strategy 2) in addition transmitted their vital signs and other data weekly by telephone. Patients assigned to strategy 2 also	Usual care	12 months

						in heart failure (HF) patients was evaluated.	made monthly 24-hour cardiorespiratory recordings; however, these data were not made available to the clinical team. The third group (strategy 3) performed the same measurements as the patients in strategy 2, but the cardiorespiratory recordings were made available for clinical management. Patients in HT strategies 2 and 3 were provided with a hand-held device that continuously recorded ECG, respiration and physical activity at home and automatically transmitted the data via a modem.		
Mussi et al. (2013) [146]	RCT	101	99	200	HF LVEF 45%	To verify the effect of an educative nursing intervention composed of home visits and phone calls on patients' knowledge about the disease, self-care, and adhesion to the treatment.	The intervention group (IG) received systematic follow-up by nurses specialised in the care of HF patients through home visits (HV) after discharge from hospital and telephone contacts to reinforce guidance. During each HV, patients received information about the disease and HF education. Telephone contacts took place at intervals of 15-30 days after HV.	Usual care	3 months
Naylor et al. (2004) [147]	RCT	118	121	139	HF	To examine the effectiveness of a transitional care intervention delivered by advanced practice nurses (APNs) to elders hospitalized with heart failure.	The intervention included: a standardised orientation and training programme led by a multidisciplinary team to prepare the APNs; the use of care management strategies based on the quality-cost model; and the implementation by the APN of an evidence-based protocol on HF. The APN conducted daily visits during hospitalisation, and home visits. If a patient was re-hospitalised for any reason during the intervention period, the APN resumed daily visits to the hospital.	Usual care	52 weeks
Nucifora et al. (2006) [148]	RCT	99	101	200	HF	To evaluate the effects of a HF management programme, which included patient education and regular outpatient contact with the HF team, on re-hospitalisation and death, optimising the resources.	The intervention consisted of intensive face-to-face education prior to discharge on HF by an experienced cardiovascular research nurse. After discharge, the study nurse telephoned the patient to assess problems, promote self-management and reinforce education. The nurse also assessed the patient's compliance and	Usual care	15 days, 1 and 6 months

							adherence to treatment, using a pre-filled questionnaire. Patients were encouraged to telephone the nurse in case of worsening.		
Oddone et al. (1999) [149]	RCT	222	221	443	HF	To determine whether enhanced access to primary care affects the diagnostic evaluation, pharmacologic management, or health outcomes of patients hospitalized with congestive heart failure (CHF).	The hospital part of the intervention included education provided by the nurse. The GP visited the patient in hospital before discharge and developed a list of problems and a treatment plan with the nurse. After discharge, patients received a phone call from the nurse to assess potential difficulties with medication or medical regimes and to identify health problems. The patient also received an appointment with the general practitioner and nurse.	Usual care	6 months
Ong et al. (2016) [150]	RCT	715	722	1437	HF	To evaluate the effectiveness of a care transition intervention using remote patient monitoring in reducing 180-day all-cause readmissions among a broad population of older adults hospitalized with HF.	The Better Effectiveness After Transition - Heart Failure (BEAT-HF) intervention consists of three components: heart failure education before discharge, periodic telephone coaching and home telemonitoring. Pre-discharge health education is conducted with the "teach-back" approach by the practice nurse and guides the patients through a brochure. Telephone nursing care then takes place on a weekly basis during the first month after discharge.	Usual care	3 months
Paradis et al. (2010) [151]	RCT	15	15	30	HF	To evaluate the preliminary effect of a motivational interviewing based on the stages of change (MISC) intervention on HF patients' self-care behaviours.	The intervention consisted of an initial face-to-face meeting prior to discharge with a trained nurse in which patients were asked to identify one behaviour they would like to change out of 5 proposed. Subsequently, two telephone interviews took place in which the nurse, following an algorithm, assessed the patient's level of conviction and confidence and the stage of change.	Usual care	6 months
Pekmezaris et al. (2012) [152]	RCT	84	84	168	HF	To study the impact of remote patient monitoring (RPM) upon the most frequent diagnosis in hospitalized patients over 65 years of age heart failure (HF).	Patients in the RPM groups received a combination of live nursing visits and RPM visits. A patient video station including screens, microphones and accessories was provided. The nurses contacted the patients from their desks at the home care agency	Usual care	3 months

							and were able to observe the patients while they weighed themselves with the scales, listened to chest sounds with the built-in stethoscope and checked their blood pressure with the cuff.		
Phillips et al. (2005) [153]	Meta-regression analysis	464	485	949	HF	To determine whether a hierarchy of effectiveness exists with respect to complexity of published protocols of heart failure (HF) disease management (DM) incorporating specialist nurse-led HF clinics.	NA	Usual care	NA
Postmus et al. (2011) [154]	RCT	340 basic support<br>344 intensive support	339	1027	HF preserved LVEF	To examine the effects of a nurse-led disease management program in a sufficiently large population with an assumed relatively high event rate. To determine the effects of 2 levels of intensity in disease management programs on the combined end points of death and readmission to the hospital.	Patients in the basic support group were visited by the HF nurse at the outpatient clinic and behavioural strategies were used to improve adherence. Patients in the intensive support group additionally received monthly contact with the nurse and multidisciplinary counselling provided by a physiotherapist, a dietician, and a social worker. The materials used included a patient diary, brochures on HF and samples of reduced-sodium food condiments.	Usual care	18 months
Powell et al. (2010) [155]	RCT	451	451	902	HF LVEF ≤40%	To determine the value of self-management counselling plus HF education, over HF education alone, on the primary endpoint of death or HF hospitalization.	The treatment of the self-management plus education arm involved HF education through group meetings and counselling to help patients develop mastery of problem-solving skills. HF management handouts were given at each meeting. Patients randomised to the education arm received the same HF information leaflets but delivered by mail. To ensure receipt and to check understanding, a study co-ordinator contacted the patient by telephone within 2-3 days of each mailing.	Usual care	1 year
Rainville et al. (1999) [156]	RCT	17	17	34	HF	Demonstration project intended to improve functional health status of patient with HF and reduce their risk of rehospitalization through the implementation of a	A pharmacist reviewed the pathology and pharmacotherapy of HF, weight monitoring and risk changes for heart failure and coronary heart disease with the patients in the intervention group. An information leaflet, a videotape,	Usual care	90 days

						pharmacist coordinated clinical service based in an acute care facility	a weight record booklet, and a medication diary were provided. After discharge, a pharmacist contacted patients in the control group by telephone to inquire about possible readmissions. A pharmacist called the intervention group and inquired about possible readmissions, answered questions from the intervention patients, reinforced the information and provided a telephone number for further assistance.		
Reilly et al. (2015) [157]	RCT	70	64	134	HF II–IV and type II DM	To conduct a prospective cost-effective analysis of a randomized clinical biobehavioral trial (Quality HF-diabetes) focused on improving comorbid self-care for persons with both HF and diabetes.	Participants in the intervention group participated in a personalised educational and counselling session. Motivational messages focused on the role of HF-DM patients in self-care, self-monitoring of glucose, weight and symptoms. At 48-72 hours, the nurse made a home visit to review glucose and weight information and the participant's interpretation of the data, followed by follow-up telephone calls.	Usual care	6 months
Rich et al. (1995) [158]	RCT	142	140	182	HF	To determine whether a multidisciplinary approach to treatment could significantly reduce the rate of readmission for elderly patients with heart failure at high risk.	The study treatment consisted of intensive education on congestive heart failure and its treatment by a nurse experienced in cardiovascular research, using an educational booklet; dietary instructions given by a dietician; consultation with social services personnel; medication analysis by a geriatric cardiologist; intensive follow-up after discharge through the hospital's home care services, supplemented by home visits and telephone contact.	Usual care	90 days
Rich et al. (1993) [159]	RCT	49	49	98	HF	To determine the feasibility and potential impact of a nonpharmacologic multidisciplinary intervention for reducing hospital readmissions in elderly patients with congestive heart failure.	The study intervention consisted of four components: intensive education on heart failure and its treatment, a detailed medication review with specific recommendations to improve compliance and reduce adverse effects, early discharge planning, and enhanced follow-up through home care and telephone contact.	usual care	1 year

Riegel et al. (2006) [160]	Mix method pre post test	NA	NA	24	HF	To describe a motivational counselling intervention and the mechanisms by which it influenced HF self-care.	A team of experts trained an experienced nurse in the motivational approach and family counselling. The participants received home visits from the APN over the course of three months. Most patients also received a follow-up phone call at the end of the interaction. The home intervention sessions were audio-recorded, transcribed verbatim, analysed by an investigator, and supplemented with field notes and personal accounts by the APN who delivered the intervention.	NA	3 months
Riegel et al. (2002) [161]	RCT	130	228	358	HF I-IV	To assess the effectiveness of a standardized telephonic case-management intervention in decreasing resource use in patients with chronic heart failure.	Patients were provided with telephone case management by a registered nurse using a decision support software programme designed to emphasise factors previously shown to predict hospitalisation in people with HF. The frequency of telephone contact was software-driven or according to the judgement of the case manager. Printed educational material was sent to patients monthly. Automatic reports produced by the software were sent to the physicians.	Usual care	3 and 6 months
Riegel et al. (2006) [162]	RCT	69	65	134	HF	To test the effectiveness of telephone case management in decreasing hospitalizations and improving health-related quality of life (HRQL) and depression in Hispanics of Mexican origin with HF.	Telephone case management was standardised using a decision support software programme. The software guides the nurse case manager in assessing factors previously shown to predict HF hospitalisation and in teaching patients' self-care skills. The intervention focused on education, monitoring and guidance. The intervention group was contacted by telephone within 5 days of hospital discharge and thereafter at a frequency guided by the software and the nurse case manager's judgement. The nurse case managers telephoned the doctors as needed and sent reports on the patients' progress at regular intervals.	Usual care	6 months

Ruschel et al. (2018) [163]	Cost-effectiveness study within a randomized trial.	123	129	252	HF LVEF ≤45%	To estimate the cost-effectiveness of a nurse-led home visit (HV) intervention as compared with the standard HF management, within a randomized clinical trial.	After hospital discharge, patients were randomly allocated to receive specialist nurse-led home visits and telephone calls or usual care (return to the outpatient clinic) over 6 months. Visiting nurses provided guidance on disease awareness, self-care practices, early recognition of the signs and symptoms of decompensation and decision making in the event of decompensation, annual immunizations, and the importance of adherence to a low-sodium diet, fluid restriction, and medications.	Usual care	6 months
Sales et al. (2013) [164]	RCT	70	67	137	HF	To evaluate the effectiveness of using trained volunteer staff in reducing 30-day readmissions of congestive heart failure (CHF) patients.	The patients in the intervention arm received instructions from the volunteer staff, including training before discharge from the hospital, a discharge sheet and follow-up phone calls after discharge. Before being discharged from the hospital, patients received a visit from one of the volunteers to receive further information about their heart failure condition and a treatment and management plan. patients received weekly follow-up phone calls for one month to reinforce the discharge instructions.	Usual care	30 days
Schofield et al. (2005) [165]	Prospective cohort study	NA	NA	92	HF II–IV LVEF <40%	To describe the early outcomes of a study before and after enrolment into the HF home-telehealth program, but without randomization or controls.	An in-home telehealth message device was provided to the patient at enrolment, and patients received daily HF-specific education via the nurse coordinator and/or the device throughout their continuum of care. Biweekly interdisciplinary team rounds were held to discuss patient status and vital signs/symptom reports.	NA	6 months
Schwarz et al. (2008) [166]	pilot study	51	51	102	HF Framingham	To examine whether telemonitoring by an advanced practice nurse reduced subsequent hospital readmissions, emergency department visits, costs, and risk of hospital readmission for patients with HF.	Patients in the intervention group received the Cardiocom EHM system. The RN data collector placed a scale in the participants' homes and connected via telephone line to a hospital computer system. The EHM measured weight on a daily basis. The device's display asked participants to answer questions about wheezing, coughing, fatigue, bloating, chest discomfort, urination, exercise,	Usual care	3 months

							dizziness, medication use and sodium intake. The computer stored each patient's electronic health record and automatically displayed clinical changes. When participants had measurements outside the prescribed parameters, the monitoring nurse called the caregiver to further assess the situation.		
Seto et al. (2012) [167]	Qualitative	NA	NA	22	HF	To provide in-depth insight into the effects of telemonitoring on self-care and clinical management, determine the features that enable successful heart failure telemonitoring.	Semi-structured interviews were conducted with 22 patients with heart failure who had been using the telemonitoring system for 6 months. The cardiologists and nurses who had interacted with the telemonitoring system were interviewed.	NA	NA
Seto et al. (2012) [168]	RCT	50	50	100	HF Amb C	To investigate the effects of a mobile phone-based telemonitoring system on heart failure management and outcomes.	Participants in the telemonitoring group used the telemonitoring system for 6 months to take daily weight and blood pressure readings as well as single-lead electrocardiograms (ECG). They were asked to answer daily questions about morning symptoms on a mobile phone. The cardiologists and nurses in the clinic handled the reports and performed interventions. If the measurements were outside the target range or if the patient reported symptoms via the mobile phone, alerts were sent by e-mail to the cardiologist's mobile phone.	Usual care	6 months
Shearer et al. (2007) [169]	RCT	45	45	90	HF	To examine the effects of a telephone-delivered empowerment intervention (EI) on clinically and theoretically relevant outcomes in patients with HF, including purposeful participation in goal attainment, self-management of HF, and perception of functional health.	The intervention group received a telephone-delivered EI that provided them support and information designed to facilitate their recognition that they could purposefully participate in setting and attaining valued goals, self-manage their HF, and improve their functional health.	Usual care	12 weeks

Shively et al. (2012) [170]	RCT	43	41	84	HF III	Examine whether HF outcomes be improved by increasing patient engagement in care and capabilities for self-care management. To determine the efficacy of a patient activation.	The activation/Heart PACT intervention was based on the activation theory and measurement described and defined by Hibbard and colleagues. The intervention was a 6-month programme developed to improve self-management and was administered by experienced nurses. Each participant met with the intervention nurses for 6 sessions, either by telephone or in person. During these meetings health behaviour goals were discussed, progress towards the goals was reinforced, barriers were addressed, and questions were answered.	Usual care	6 months
Sisk et al. (2006) [171]	RCT	203	203	406	HF	To compare the effects of a nurse-led intervention focused on specific management problems versus usual care among ethnically diverse patients with systolic dysfunction in ambulatory care practices.	In the nursing management intervention, one of the three specialist nurses met once with each patient and carried out an educational intervention. The nurse also acted as a bridge between the patient and the doctor to discuss medication and to organise changes to prescriptions and examinations. An internist monitored the work of the nurses and a cardiologist provided supervision if necessary. The nurses were bilingual and conducted follow-up calls.	Usual care	12 months
Smeulders et al. (2010) [172]	RCT	186	131	317	HF II–III LVEF <40%	To assess the effects of the Chronic Disease Self-Management Programme (CDSMP) on psychosocial attributes, self-care behaviour and quality of life among patients with CHF who experienced slight to marked limitation of physical activity.	The CDSMP is a self-management programme consisting of six weekly group sessions of two and a half hours each. It incorporates four strategies to increase expectations of self-efficacy: skills mastery, reinterpretation of symptoms, modelling, and social persuasion. The CDSMP classes were conducted by a cardiology nurse ('professional leader') and a heart failure patient ('peer leader'), both trained in the CDSMP protocol.	Usual care	1 year
Smeulders et al. (2010) [173]	RCT	186	131	317	HF II–III	Gain insight into which CHF patients benefited most from The Chronic Disease Self-Management Programme (CDSMP) in terms of cardiac-specific quality of life by	Self-management programme (CDSMP) in addition to usual care, which addresses the problems of daily management of patients with chronic diseases. The CDSMP sessions were taught by a trained cardiology nurse and a CHF patient.	Usual care	6, 12 months

						conducting additional subgroup analyses.			
Soran et al. (2008) [174]	RCT	160	155	315	HF LVEF < 40%	To assess the impact of a computer-based home disease management program (Alere DayLink HF Monitoring System [HFMS]) on the clinical outcomes of Medicare beneficiaries with HF who were elderly, women, and non-white males who received the care from a community-based primary care practitioner.	The intervention consisted of HFM in addition to standard care. HFMS consists of a home-based disease management programme to monitor and detect early signs and symptoms of heart failure using telecommunication equipment. The system includes an electronic scale and a system with symptom response monitors connected to a computerised database with trained nurses. Patients were instructed to weigh themselves and answer questions on the scales. Each patient's primary care physician was responsible for selecting the monitoring parameters. Changes in weight or changes in symptoms were reported to the primary care physician by the nursing staff.	Usual care	6 months
Stewart et al. (1998) [175]	Prospective cohort study	49	48	97	HF II-IV	To examine the effect of a home-based intervention (HBI) on readmission and death among "high-risk" patients with congestive heart failure discharged home from acute hospital care.	Prior to discharge, patients assigned to HBI were visited by the study nurse and counselled regarding compliance with the treatment regimen and the reporting of clinical problems. One week after discharge, these patients were visited at home by the study nurse and the study pharmacist. The pharmacist assessed the knowledge of the prescribed medication and the degree of compliance. The patients were further evaluated by the nurses for clinical worsening or adverse effects of the medication. All patients' GPs were contacted by the practice nurse to inform them of the home visit and discuss corrective actions.	Usual care	6 months
Stewart et al. (2002) [176]	RCT	149	148	297	HF LVEF<45 %	Prospective evaluation of the long-term effects of a post discharge home-based, multidisciplinary intervention (HBI) on a primary end point of death	In both studies, patients assigned to HBI received a structured home visit within 7-14 days after discharge. In the original study, this visit was performed by a nurse and a pharmacist. In the later study, the	Usual care	3-6 years

						or unplanned readmission in a cohort of typically older patients with CHF who participated in 2 randomized studies.	home visit was performed by a qualified cardiac nurse. Only in the first study did patients receive additional education about their condition and treatment, and only in the second study did a small number of patients receive repeated home visits. The fundamental aim of HBI is to optimise the management of the patient's chronic disease state and to facilitate the early recognition and treatment of potential problems.		
Stewart et al. (2002) [177]	RCT	49	48	97	HF II-IV LVEF ≤55%	To examine the effect of a home-based intervention (HBI) on the frequency of unplanned readmissions plus out-of-hospital deaths for 6 months among "high-risk" patients with CHF discharged from acute hospital care.	Prior to discharge, patients assigned to an HBI were visited by the study nurse and counselled on compliance with the treatment regimen. One week after discharge, these patients were visited at home by the study nurse and the study pharmacist. Upon arrival, the study pharmacist carried out an assessment of the knowledge of the prescribed medication and the degree of compliance. Patients who demonstrated poor medication knowledge or compliance received corrective counselling, routine reminders, introduction of a weekly medication container, incremental monitoring by caregivers and referral to a community pharmacist for more regular review later.	Usual care	6 months
Stewart et al. (1999) [178]	Prospective cohort study	100	100	200	HF II-IV LVEF 55%	To test the null hypothesis that there would be no difference in the frequency of unplanned readmission plus out-of-hospital death, during a minimum of 6 months of follow-up, among patients with chronic CHF discharged home after acute admission between those exposed or not exposed to a CHF-specific, multidisciplinary, home-based intervention in addition to usual care.	Patients assigned to the home intervention received a home visit by a qualified cardiology nurse after discharge, in addition to normal therapy. During the visit, the nurse assessed the patient's clinical progress, performed a physical examination, and evaluated adherence to therapy, the extent of psychosocial support and the use of available community resources. Based on this assessment, patients and their families received a combination of counselling and improvement strategies. The nurse also recommended possible changes in pharmacotherapy. The patients were contacted by telephone.	Usual care	6 months

Stro¨mber get al. (2003) [179]	RCT	52	54	160	HF II - IV	To evaluate the effect of follow-up at a nurse-led heart failure clinic on mortality, morbidity and self-care behaviour for patients hospitalised due to heart failure for 12 months after discharge.	Patients in the intervention group were followed at a heart failure clinic run by specially trained and experienced cardiology nurses, who were delegated the responsibility for making medication changes according to the protocol. During the visit, the nurse assessed the status and whether the heart failure treatment was optimised, provided heart failure education and social support to the patient and his family.	Usual care	12 months
Thompson et al. (2005) [180]	RCT	48	58	106	HF LVEF <45%	To determine the effectiveness of a hybrid program of clinic plus home-based intervention (C+HBI) in reducing recurrent hospitalisation in CHF patients.	Patients assigned to HBI+C were visited by the practice nurses experienced in the management of heart failure before discharge and received a home visit within 10 days after hospital discharge. During the ward visit, patients received information about their condition, medication, and the telephone number to contact the practice nurse. During the home visit, patients were instructed on the recognition of symptoms, their management and lifestyle issues and received a clinical examination. The patients attended a monthly heart failure clinic run by a nurse for at least 6 months. During the clinical visits, the patients received a comprehensive educational package, a clinical evaluation and laboratory tests.	Usual care	6 months
Triller et al. (2007) [181]	RCT	77	77	154	HF	To evaluate the effect of pharmaceutical care services for home care patients with heart failure on death and rehospitalization rates.	The pharmaceutical care services consisted of an initial comprehensive medication assessment at home and two follow-up visits. The follow-up visits were conditional on the patient continuing to receive the services of the visiting nurse. The pharmacist interacted with the prescribing physicians on behalf of the patients, if necessary.	Usual care	3 months

Tsuchihashi-Makaya et al. (2011) [182]	RCT	78	78	156	HF II—IV	To determine the effectiveness of interventions, as compared to that of usual care, on psychological status, including depression and anxiety, on all-cause death, cardiac death, sudden cardiac death, readmission due to decompensated HF, and QOL in HF patients.	Home-based disease management group consisted of following interventions:<br>1. Home visit by nurse<br>2. Telephone follow-up by nurse<br>3. Routine follow-up by cardiologist	Usual care	12 months
Tsuchihashi-Makaya et al. (2013) [183]	RCT	79	82	161	HF	To determine the effects of home-based disease management on the psychological status of patients with HF.	Enrolled patients received comprehensive education at discharge using a brochure provided by a cardiologist, nurse, dietician, or pharmacist. The booklet provides knowledge and information on pathophysiology, medical treatment, diet, physical activity, lifestyle modification, self-measurement of body weight, self-monitoring of worsening HF and emergency contact methods. The home disease management programme consists of a home visit and telephone follow-up counselling by the nurse.	Usual care	12 months
Tsuyuki et al. (2004) [184]	RCT	140	136	276	HF	To evaluate the effect of a disease management program on clinical and economic outcomes in patients with HF.	Patient support program (PSP) consisted of education about HF, self-monitoring, adherence aids, newsletters, telephone hotline, and follow-up at 2 weeks, then monthly for 6 months after discharge.	Usual care	6 months
van der Wal et al. (2010) [185]	Qualitative	NA	NA	15	HF	To explore patients' reasons and motivations for compliance with medication, sodium-restricted diet, fluid restriction, and daily weighing, including an evaluation of how patients manage the heart failure regimen in daily life.	The patients in this study had recently finished participating in the COACH study. Two independent researchers classified the interviews into broad themes, partly based on HBM.	NA	18 months
van Veldhuisen et al. (2011) [186]	RCT	168	167	335	HF II–IV LVEF <35%	Determine whether measuring intrathoracic impedance with an implanted device with an audible patient alert can detect increases in pulmonary fluid retention early, hypothesising that	The patients had undergone implantation of an implantable cardioverter-defibrillator alone or with cardiac resynchronisation therapy. A fluid detection algorithm was used in the access arm to alert the patient if a certain level of impedance drop occurred. A	Usual care	6 months

						early intervention can prevent hospitalisations and influence outcomes.	management strategy was used with access to all device-based diagnostic information. Whenever a device alarm occurred, the protocol always required a patient-physician contact.		
Wakefield et al. (2008) [187]	RCT	52	49	148	HF	To evaluate the efficacy of a telehealth-facilitated post discharge support program in reducing resource use in patients with heart failure.	A telehealth-facilitated intervention, using telephone or videophone. Intervention subjects were given a symptom review checklist to help them report HF-related symptoms, a scale, blood pressure cuff, and tape measure and were instructed to measure daily weights, blood pressure, and ankle circumference to monitor fluid accumulation. Study nurses reviewed the discharge plan during the first intervention contact and reinforced it during subsequent contacts. Study nurses also employed following strategies to improve subjects' compliance with prescribed treatment plans: behavioral skill training strategies (e.g., reviewing skills for monitoring blood pressure, and recommending use of a grocery list to purchase low-sodium food), self-monitoring strategies (e.g., keeping a daily record of weight and food intake), self-efficacy enhancement strategies (use of community support groups; encouragement and feedback; and realistic goal setting), external cognitive strategies encouraging use of aids such as meal plans and medication organizers.	Usual care	3 months
Walsh et al. (2012) [188]	RCT	94	61	155	HF	Understand whether the implementation of a performance improvement system in outpatient practices with Electronic Health Record systems (EHR) may better demonstrate the value of EHR in improving quality.	The intervention included a clinical decision support toolkit based on guidelines, educational materials, practice-specific data reports, quality of care referral reports, and structured training and collaboration opportunities. A web-based system provided quality of care reports for each practitioner that included benchmark comparisons with cardiology specialists at regional and national levels. Participating practitioners were encouraged to	Usual care	24 months

							participate in bi-monthly educational and collaborative web-based seminars and to continuously evaluate, refine, and reassess the delivery of care during the intervention phase of the study.		
Weintraub et al. (2010) [189]	RCT	93	95	188	HF	To assess the incremental effect of automated health monitoring (AHM) technology over and above that of a nurse directed heart failure (HF) disease management program.	Subjects randomised to the intervention arm received an AHM system. The monitoring system included a device that measured and communicated body weight, blood pressure and heart rate to a central server, and an interactive communication device designed to transmit textual information regarding symptoms, functional status and medication compliance. If the measured data exceeded pre-set limits, the HF nurse called the patient to discuss the deviation and initiate an intervention.	Usual care	3 months
Wierzchowiecki et al. (2006) [190]	RCT	80	80	160	HF	To determine whether multidisciplinary care (MDC) for patients with CHF has an influence on mortality, rate of rehospitalization, quality of life (QoL) and self-care (SC) during a one-year study period.	The patients in the MDC group had follow-up visits at the Heart Failure Clinic which included consultation with the cardiologist, HF nurse, physiotherapist, and psychologist. Patients and their general practitioners also had the opportunity of a telephone consultation by the HF nurse and the cardiologist. For patients with advanced HF who could not go to the heart failure clinic, the HF nurse made a home visit. Between visits, patients were followed up by their general practitioners. Once a month, the clinic's doctors and cardiologists held a lecture for patients and their families on the nature, aetiology, diagnosis and treatment of heart failure. The HF nurse in charge gave a one-to-one lecture in the clinic, at the patient's home or by telephone.	Usual care	12 months

Wongpiriyayothar et al. (2011) [191]	RCT	11	11	22	HF II-III	To examine the effects of coaching using the telephone (CTP) on dyspnea and physical functioning among persons with HF.	In the experimental group, the researcher provided coaching using CTP; participants received a single face-to-face patient education session at the outpatient clinic and a brochure on HF. Telephone coaching was provided by the research nurse at least twice a week for three consecutive weeks. The research nurse coached individual participants by telephone on self-monitoring, symptom management and adherence to prescribed medication, restricted diet, fluids, and exercise.	Routine hospital or clinic assessment and education	4 weeks
Woodend et al. (2008) [192]	RCT	124	125	249	HF II	To determine whether tele home monitoring of patients with cardiac disease at high risk of readmission would reduce hospital readmissions, improve functional status, and improve quality of life over usual care.	The intervention consisted of 3 months of video conferencing with a nurse for the purpose of monitoring and educating patients with HF and angina. Weight, blood pressure and electrocardiogram were transmitted via electronic detectors to a central station containing the patient's electronic medical record.	Usual care	3 months
Wright et al. (2003) [193]	RCT	100	97	197	HF	To evaluate the use of a heart failure diary and a schedule of daily self-weighing for patients enrolled in an integrated, comprehensive, out-patient heart failure management program, the Auckland heart failure management study (AHFMS).	Patients randomised to the heart failure programme underwent an outpatient clinical follow-up, followed by scheduled visits. Clinical appointments included HF counselling and education by a specialist nurse, optimisation of therapy by a specialist physician and liaison with primary healthcare providers. A heart failure diary was handed out, which included a list of medications, contact details for the outpatient clinic, an appointment schedule, and a daily weighing schedule to be completed.	Usual care	12 months
Yu et al. (2005) [194]	Prospective cohort study	/	/	34	HF III-IV	To evaluate an implantable system capable of measuring intrathoracic impedance to identify potential fluid overload before heart failure hospitalization and to determine the correlation between intrathoracic impedance and standard	After implantation of the device, patients entered the chronic follow-up phase, which lasted up to 2 years. Patients were seen monthly by a physician for the first year and as needed for disease management during the second year. The heart failure nurse assessed compliance with medication and symptoms with weekly telephone contact for the first year. Intrathoracic impedance	NA	The mean follow-up period was 20±8.4 months.

						measures of fluid status during hospitalization.	was measured automatically every 6 hours and subsequently retrieved from a monitor for analysis.		
Yu et al. (2015) [195]	RCT	90	88	178	HF	To determine the effect of nurse-implemented transitional care (TC) on readmission and mortality rates in Chinese individuals with chronic heart failure CHF) in Hong Kong.	The TC programme implemented by the nurse comprised several care components, including pre-discharge visits, home visits, intensive telephone follow-up and telephone access to a cardiology nurse. The nurse conducted a pre-discharge visit with the TC group to assess health status, cultural beliefs, self-care practices and post-discharge needs. After hospital discharge, he conducted two weekly home visits. He provided customised educational and support interventions. He also helped identify appropriate community care services. Subsequently, he made telephone calls; participants had telephone access to the cardiology nurse.	Usual care	6 months
